# PIM1 instigates endothelial-to-mesenchymal transition to aggravate atherosclerosis

**DOI:** 10.7150/thno.102597

**Published:** 2025-01-01

**Authors:** Zhiwei Xue, Mengtao Han, Tao Sun, Yanzhao Wu, Wenchen Xing, Feiyu Mu, Zhihan Zhang, Junzhi Liu, Xiangjun Liang, Lu Ling, Jian Wang, Jiwei Wang, Xingang Li, Bin Huang, Donghai Wang

**Affiliations:** 1 Department of Neurosurgery, Qilu Hospital, Cheeloo College of Medicine and Institute of Brain and Brain-Inspired Science, Shandong University, Shandong, China.; 2 Shandong Key Laboratory of Brain Health and Function Remodeling, Jinan 250012, China.; 3 Department of Neurosurgery, Qilu Hospital of Shandong University Dezhou Hospital, Dezhou, China.; 4 Jinan Microecological Biomedicine Shandong Laboratory and Shandong Key Laboratory of Brain Function Remodeling, Jinan, China.; 5 Department of Biomedicine, University of Bergen, Jonas Lies Vei 91, 5009, Bergen, Norway.

**Keywords:** Atherosclerosis, Endothelial-to-mesenchymal transition, PIM1, Nuclear translocation, Max-40279

## Abstract

**Background:** Endothelial-to-mesenchymal transition (EndMT) is a cellular reprogramming mechanism by which endothelial cells acquire a mesenchymal phenotype. Endothelial cell dysfunction is the initiating factor of atherosclerosis (AS). Increasing evidence suggests that EndMT contributes to the occurrence and progression of atherosclerotic lesions and plaque instability. However, the mechanisms leading to EndMT in atherosclerotic plaques' microenvironment are poorly understood.

**Methods:** Single-cell sequencing data of atherosclerotic plaques in mice fed with high-fat diet for different time periods were analyzed. Using quantitative polymerase chain reaction, western blotting, and immunohistochemistry, we demonstrated that the expression of PIM1 in ox-LDL stimulated endothelial cells and in human and mouse atherosclerotic lesions. *ApoE*^-/-^ C57 mice were injected recombinant adeno-associated virus serotype 9 through tail vein to explore the role of PIM1 in atherosclerosis. Co-immunoprecipitation (Co-IP) was used to verify the substrates of PIM1. Hematoxylin and eosin (H&E) staining, Oil Red O staining, and Masson's trichrome staining were used to assess the size of atherosclerotic plaques, lipid content, and collagen fiber content, respectively.

**Results:** PIM1 expression in endothelial cells increased with the progression of AS *in vivo*. Endothelial cell-specific PIM1 knockdown negatively regulated atherosclerosis progression and the EndMT process. Knockdown of PIM1 in endothelial cells *in vitro* attenuated ox-LDL-induced EndMT. This process was primarily due to the reduction of PIM1, which led to decreased phosphorylation of NDRG1 at Ser330, and subsequently, reduced NDRG1 nuclear translocation. Consequently, the interaction between NDRG1 and PTBP1 was affected, ultimately impacting the mRNA levels of Vimentin, ZEB1, Slug, Snail, N-Cadherin, TAGLN, and α-SMA. The small molecule Max-40279 could inhibit NDRG1 phosphorylation at Ser330 and suppress EndMT.

**Conclusion:** Our findings revealed the PIM1/P-NDRG1(S330)/PTBP1/EndMT axis as a critical factor promoting AS progression and could generate new strategies to prevent AS.

## Introduction

Endothelial-to-mesenchymal transition (EndMT) is an intricate cellular process of endothelial cell reprogramming and redifferentiation, characterized by endothelial cell detachment and migration away from the endothelium and, to varying extents, decreasing endothelial properties and acquiring mesenchymal features. EndMT is primarily characterized by cell morphology changes and cell markers alterations. For instance, the expression levels of endothelial cell markers (CD31, VE-Cadherin, ZO-1) decrease, while conversely, the expression levels of mesenchymal cell markers (ZEB1, N-Cadherin, α-SMA, Vimentin, TAGLN, SLUG, SNAIL) increased 1-3. EndMT has been recognized as a crucial pathophysiological mechanism involved in sustaining chronic vascular inflammation and contributing to the pathogenesis of several diseases, including malignant diseases 4, fibrotic diseases 5, pulmonary arterial hypertension 6, AS 7, diabetes mellitus 8, cavernous malformations 9, and fibrodysplasia ossificans progressive 10. Numerous triggers, including cytokines, growth factors, oxidative stress, and inflammatory signals, can cause EndMT, and various signaling pathways, most notably the TGF-β pathway, have been linked to EndMT 1, 11.

AS is a progressive vascular disease characterized by the accumulation of lipid, fibrous, and cellular components within the thickened intima of arteries, forming plaques. Endothelial dysfunction is considered the initial event in the pathogenesis of AS 12-15, and most commonly occurs at branched and curving areas of the vasculature, where the vessels are exposed to disturbed blood flow patterns. Endothelial cells experience oscillatory low-amplitude shear stress (OSS) in these locations, leading to endothelial dysfunction and reprogramming. This includes increased inflammation, permeability, apoptosis, cell proliferation, EndMT, and endothelial cell transformation into immune cells 16, contributing to plaque formation. Once plaques form, the inflammatory, oxidative stress, and high TGF-β environment within the plaques further promote EndMT, creating a vicious cycle 17, 18. Recent studies have shown that nearly one-quarter of Chinese adults have an increased carotid intima-media thickness (cIMT) or carotid plaque (CP) 19. For this large population with these conditions, in-depth exploration of the pathogenesis and the search for effective treatment methods are crucial for preventing and managing cardiovascular and cerebrovascular diseases.

Here, we analyzed single-cell sequencing data of atherosclerotic plaques from mice at different stages and found that PIM1 expression in endothelial cells was increased, particularly in advanced plaques, including those from mice on a high-fat diet (HFD) for 26 weeks. PIM1 belongs to the proviral insertion site in Moloney murine leukemia virus, a family of short-lived serine/threonine kinases 20. Numerous studies have shown that PIM1 can cause pulmonary fibrosis and promote the progression of various tumors by promoting epithelial-to-mesenchymal transition (EMT) 21-25, which is closely related to EndMT 1. The PIM kinase has also been shown to play an important role in cardiovascular diseases 26. Based on this body of data, we speculated that expression of PIM1 may govern EndMT. Furthermore, we hypothesized that by modulating EndMT, PIM1 may participate in maintaining endothelial cell homeostasis and advancing AS and plaque instability.

## Methods

### Data availability

The authors declare that all supporting data are available in the article and Supplemental Material. The research materials listed in the Methods are included in the Major Resources Table in the Supplemental Material.

FASTQ files of atherosclerotic plaques in mice with atheroprone backgrounds (*Ldlr*^-/-^) were obtained from the NCBI Gene Expression Omnibus (GEO) database under the accession number GSE155513. The RNA-seq data are available in the GEO database (accession: GSE118446, GSE43292, GSE206927).

### Patients and samples

This study was approved by the ethics committee of Qilu Hospital (KYLL-202111-037-1), informed consent was obtained from all patients prior to the procedure. Patients undergoing CEA for carotid atherosclerotic stenosis or occlusion were enrolled in the study between December 2020 and January 2023. All patients were diagnosed, and other etiologies were excluded by Doppler ultrasound, computed tomography angiography (CTA), magnetic resonance imaging or angiography, and digital subtraction angiography (DSA). The minimum age limit was 18 years. Data on the clinical and demographic characteristics of the participants were gathered from before CEA. The clinical characteristics of the patients are reported in Supplementary Table S1.

### Animals

All animal experimental protocols used in this study were approved by the ethics committee of Qilu Hospital, Shandong University (Jinan, China; approval number: DWLL-2023-080). Mice were housed in the Qilu Hospital of Shandong University animal facilities and exposed to light on 12 h cycles in a humidity- and temperature-controlled environment with no pathogenic microorganisms. Endo-*Pim1*^KD^ mice were established by recombinant adeno-associated virus serotype 9 (AAV9). Eight-week-old male *ApoE*^-/-^mice were microinjected with either the endothelial cell-specific control shRNA-AAV9 (AAV-ICAM2-empty vector) or *Pim1* shRNA-AAV9 (AAV-ICAM2-sh*Pim1*; 5×10^11^ vg; Vigene Biosciences, Jinan, China) via the tail vein. The sequence for sh*Pim1* was 5ʹ-CCGAUAGUUUCGUGCUGAU-3ʹ. After 4 weeks, HFD was administered for 16 weeks to develop atherosclerosis.

### Cell culture

Human Vein Endothelial Cells (HUVECs) and Mouse Aortic Endothelial Cells (MAECs) were provided as a gift from the Department of Cardiology, Qilu Hospital of Shandong University, and cultured at 37 °C in 5% CO_2_ in Endothelial Cell Medium (Catalog No. 1001, Science Cell) supplemented with 15% fetal bovine serum (FBS) (Catalog No. 0025, Science Cell), 1% Endothelial Cell Growth Supplement (ECGS, Catalog No. 1052, Science Cell), and 1% penicillin/streptomycin solution (P/S, Catalog No. 0503, Science Cell). Mouse Primary Lung Endothelial Cells (MPLECs) were obtained from *ApoE*^-/-^ Endo-*Pim1*^KD^ C57 mice or *ApoE*^-/-^ Endo-Control C57 mice. For each batch of MPLECs, 4 mice were anesthetized by intraperitoneal injection and perfused with 10 mL ice-cold HBSS buffer (14025092, Gibco™). Lungs were removed and rinsed in Dulbecco's Modification of Eagle's Medium (DMEM) (CM10013, Macgene) with 1% anti-biotic/antimycotic (15240062, Gibco™) before being minced using a scalpel. Minced lungs were digested in 20 mL HBSS buffer containing 1% collagenase Type I (17100-017, Gibco™) for 2 hours at 37°C with constant tilting. Cells were then filtered through a 70 μm nylon filter (352350, Falcon) and washed twice before further incubated with 50μL Dynabeads™ CD31 (11155D, Invitrogen™) for 60 minutes at 4 °C on a roller. Bead-bound cells were collected using a magnet, resuspended in Endothelial Cell Medium (Catalog No. 1001, Science Cell), and cultured for at least 7 days to allow adherence and initial expansion 27. HEK293T cells were cultured at 37 °C in 5% CO_2_ in DMEM (CM10013, Macgene) supplemented with 10% FBS and 1% P/S.

EndMT was induced by incubating cells in 50 ng/mL recombinant human TGF-β2 (HY-P7119, MCE) and 200 mM H_2_O_2_ (MM0707-500ML, Shanghai Maokang Biotechnology Co., Ltd.) added to complete median days 1 and 3, as described 28.

### siRNA interference, plasmid transfection, lentivirus infection

Short interfering RNAs (siRNAs) were transiently transfected with Lipofectamine 3000 reagent (L3000150, Thermo Fisher Scientific) following the manufacturer's instructions. For plasmids, transient transfections were performed with Lipofectamine 3000 reagent (L3000150, Thermo Fisher Scientific) following the manufacturer's instructions. The lentivirus used for stable knockdown NDRG1 was purchased from OBiO Technology. Lentiviral infected cells were selected with puromycin and mRNA levels were determined using qRT-PCR. Custom-made siRNA and shRNA-lentivirus sequences are listed in Table S2 and Table S3. The plasmids and the vector used are shown in Table S4.

### Quantitative Real-Time PCR (qRT-PCR)

Total RNA was extracted using a kit from Yishan Bio (ES-RN001), according to the manufacturer's instructions. cDNA was generated from total RNA using the Hifair Ⅲ 1st Strand cDNA Synthesis SuperMix for qPCR (gDNA digester plus) (11141ES60, Yeasen). qRT-PCR was performed with Hieff qPCR SYBR Green Master Mix (No Rox) (11201ES08, Yeasen) on the Roche 480II Real-Time PCR Detection System (Roche; Basel, Switzerland). The expression of target mRNA was normalized with β-actin mRNA expression. The primer sequences used are shown in Table S5.

### Western blotting analysis

Total proteins were extracted with RIPA lysis buffer containing a cocktail of protease inhibitors and phosphatase inhibitors (P0013B, Beyotime). Cytoplasmic and nuclear extracts were prepared according to the instructions of the NE-PER® nuclear and cytoplasmic extraction kit (Pierce) (78833, Thermo Scientific). The concentration of proteins was determined with the Enhanced BCA Protein Assay Kit (P0009, Beyotime) according to the manufacturer's instructions. Equal amounts of proteins per sample (30 µg) were separated by SDS‒PAGE and then electrotransferred onto PVDF membranes (Merck Millipore; Billerica, MA, USA). Membranes were blocked for 10 min in QuickBlockTM Blocking Buffer (P0235, Beyotime), incubated with primary antibodies overnight at 4 °C, and followed by incubation with an HRP-conjugated secondary antibody (ZB-5301, ZB-2305, ZSGB-BIO) for 1 h. ECL (36208ES76, Yeasen) was utilized to detect immunoreactivity. β-actin served as a loading control. The primary antibodies used are listed in Table S6.

### Immunofluorescence (IF)

For cellular immunofluorescence staining, 2000 cells per well were seeded overnight into Slide 8 Wells (Ibidi GmbH). Treatments were added to each well on the next day, and after 72 h, each well was fixed with 4% paraformaldehyde in PBS, blocked, and permeabilized for 1 h in 1% BSA. Cells were incubated with primary antibodies overnight at 4 °C at an appropriate dilution, and subsequently for detection, with species-appropriate, fluorescently labeled secondary antibodies for 1 h at room temperature at a dilution of 1:2000. TRITC Phalloidin (40734ES75, Yeasen) was used to visualize F-actin, and DAPI (blue) (C1002, Beyotime) as a nuclear counterstain (30 min. incubation). A Leica TCS SP8 confocal microscope was used to capture the images (Leica Microsystems; Wetzlar, Germany).

For immunofluorescence staining of tissue sections, paraffin sections were incubated at 70 °C for 30 min, dewaxed in xylene and 100% ethanol, and air dried. Sections were washed three times in PBS. Antigen retrieval was then performed with EDTA Antigen Retrieval Solution (P0085, Beyotime) for 30 min at 95 °C, and the sections were allowed to cool at room temperature for 1 h. Frozen sections were directly washed three times in PBS. The sections were incubated with 3% H_2_O_2_ for 10 min and then blocked for 30 min. (0.5% Triton X-100, 1% BSA, 10% donkey serum). Incubations with primary and secondary antibodies were subsequently performed as described above. The primary antibodies used are shown in Table S6.

### Immunohistochemistry (IHC)

Paraffin sections were processed exactly as described above for IF staining. Sections were incubated with primary antibodies overnight at 4 °C at an appropriate dilution and then performed with Immunohistochemical kit (PV-9000, ZSGB-BIO) following the manufacturer's instructions. The sections were scanned, and images were captured by the WISLEAP Scanning System (WISLEAP Medical Technology Co. LTD). Staining was evaluated independently to determine the histological score according to the proportion of positively stained cells and staining intensity. The primary antibodies used are shown in Table S6.

### HE staining and quantification of lesion size

The embedded tissues were placed on a slide and were cut using a frozen slicer at the optimal cutting temperature. According to AHA guidelines, we collected cross-sections at a 6 μm distance per section on slides from the origin of the aortic valves to the ascending aorta. The left carotid artery was embedded in paraffin and sectioned, while the right carotid artery was subjected to frozen sections. Both sections were taken at the carotid bifurcation. Sections were stained with hematoxylin for 5 min, rinsed with tap water until the nuclei turned blue, and stained with eosin for 3 min. The sections were dehydrated using different concentrations of ethanol, treated with environmental transparency agent, and sealed with neutral balsam (G8590, Solarbio). Images were captured using the WISLEAP Scanning System (WISLEAP Medical Technology Co. LTD). The plaque areas were determined by the NDP.view software (2.8.24).

### Oil red O staining

The entire artery was separated and incubated in oil red O (C0158M, Beyotime) solution for 30 min and then washed with staining wash buffer. Images were captured using the Murzider surgical microscope. Frozen sections dried at room temperature were placed in oil red O for 15 min and then rinsed with staining wash buffer for 2 min. The sections were placed into a hematoxylin staining solution to stain the nuclei for 2 min and then sealed with glycerin gelatin. Images were captured by the WISLEAP Scanning System (WISLEAP Medical Technology Co. LTD). The percentage of Oil Red O positive area were determined by Image-J (1.53C) software.

### Masson staining

Frozen sections dried at room temperature were stained according to the manufacturer's protocol of masson trichrome staining kit (G1340, Solarbio). The sections were dehydrated using different concentrations of ethanol, treated with environmental transparency agent, and sealed with neutral gum. Images were captured by the WISLEAP Scanning System (WISLEAP Medical Technology Co. LTD). The percentage of collagen positive area were determined by Image-J (1.53C) software.

### RNA immunoprecipitation assay

The Magna RIP Kit (No. 17-701, Millipore) was used for the assay. Briefly, magnetic protein A/G beads pretreated with 5 µg anti-PTBP1 antibody or 5 µg Mouse IgG antibody were added to cell lysates and the samples were incubated at 4 °C under rotation overnight. Then, the beads were washed 6 times and incubated with proteinase K buffer. RNA was extracted from the immunoprecipitates and reversed transcribed. RT-qPCR products were subjected to agarose gel electrophoresis.

### Co-immunoprecipitation (Co-IP)

Co-IP was performed with the Pierce™ Classic Magnetic IP/Co-IP Kit (88804, Thermo Fisher Scientific) according to the manufacturer's instructions. The cell lysates were incubated with antibodies (listed in Table S6) or anti-IgG (3420S; Cell Signaling Technology) overnight at 4 °C, and then mixed with Protein A/G magnetic beads (88804, Thermo Fisher Scientific) and incubated for 2 h at room temperature. The proteins were eluted, separated by SDS‒PAGE, and analyzed by western blotting.

### Protein purification and Surface Plasmon Resonance (SPR)

SPR was performed with SensiQ (The Pioneer platform, ForteBio; Freemont, CA, USA). First, the SPR chip (Hiscap biosensor, ForteBio) was activated with 1 mM NiCl2, and 50 μg/mL NDRG1 protein (Ag25424, Proteintech) was immobilized on the chip. PIM1 protein (HY-P701745, MCE), PTBP1 protein (Ag28404, Proteintech), and small molecule Max-40279 (HY-145723, MCE; 500 nM) binding activities were generated with the SPR system, and the binding signal was exhibited by the response (RU) value. The data were normalized to control and analyzed with Qdat (ForteBio). Binding curves were subsequently generated.

### Transwell assays

Transwell assays were conducted in 24-well plates. The upper chamber was seeded with HUVECs, MAECs or MPLECs (3×10^4^ cells/300 µL) in FBS-free medium, and the lower chamber was filled with medium supplemented with 15% FBS. Cells that passed through the 8 µm membrane were fixed with 4% paraformaldehyde and stained with crystal violet. Brightfield microscopy images were captured, and the cells were counted.

### Scratch wound healing assay

HUVECs, MAECs or MPLECs monolayers treated as indicated in the six well plate and were scratched with a 200-μL pipette tip. Cells were washed once, and 2% serum medium was added to prevent confounding effects of FBS-induced cell proliferation. Images were taken at 0- to 12 h time points, and cell migration was determined by measuring changes in scratch area over time using Image-J (1.53C) software.

### Mass Spectrometry (MS)

Co-immunoprecipitated proteins were subjected to analysis by MS. In brief, the co-immunoprecipitated protein samples were separated by SDS-PAGE gel electrophoresis and stained with Coomassie Brilliant Blue to visualize the protein bands. The gel bands were then subjected to in-gel digestion, and the protein composition of the samples was determined using MS. The MS was conducted by Novogene Co. Ltd.

### Protein-protein docking

Protein-protein docking is a computational method used to predict the near-native structure of a protein complex based on the known three-dimensional structures of two individual proteins. Docking involves both sampling and scoring processes. In this study, protein docking was performed using the HDOCK server. HDOCK combines physical and bioinformatics-based approaches to develop efficient molecular docking algorithms and accurate scoring functions for biomolecular interactions. HDOCK samples all possible binding modes between two separate proteins. The scoring function then ranks the sampled binding modes based on their likelihood. Using the HawkDock server, the binding free energy of the two proteins was calculated using the MM/GBSA method. The binding process of the two proteins was visualized in surface representation. The binding interface of the protein-protein complex was comprehensively described and systematically analyzed using the PLIP interaction analysis platform. Additionally, interaction details were further supplemented using PyMOL.

### Statistical analyses

Statistical analyses were performed using GraphPad Prism (version 8.3; GraphPad Software, CA). The observed results are presented as mean ± SD. For independent samples, differences between two groups with equal variance were analyzed using a two-tailed Student's t-test. One-way ANOVA was used to determine differences among multiple groups with equal variance. All statistical data are derived from biological replicates. *P*<0.05 was considered statistically significant.

## Results

### PIM1 is significantly elevated in human unstable carotid atherosclerosis plaques and mouse advanced atherosclerosis plaques

**Figure 1:**
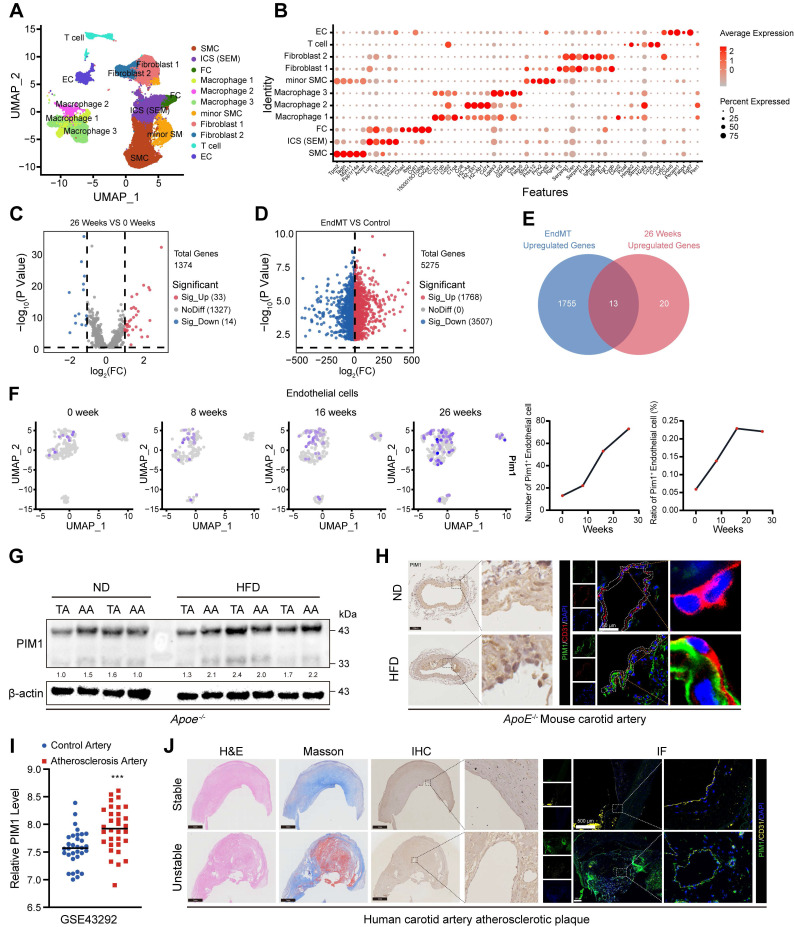
** PIM1 is significantly elevated in human unstable carotid atherosclerosis plaques and mouse advanced atherosclerosis plaques.** (A) Uniform manifold approximation and projection (UMAP) visualization of vascular cells. (B) Dot plot overview of expression of key marker genes identified for the cell types. (C) Volcano plot showing differential expression genes of endothelial cells in 0 weeks and 26 weeks. (D) Volcano plot showing differential expression genes of HUVECs in normal status and EndMT status. (E) Venn diagram showing 13 overlapping genes between genes differentially expressed in mouse atherosclerotic plaques and genes identified in HUVECs. (F) The expression level of PIM1 in endothelial cells of atherosclerotic plaques at different time points. (G) Representative Western blot images and quantification of PIM1 levels in arcus aortae and thoracic aorta from normal diet (ND) and high fat diet (HFD) mice. (H) Representative immunohistochemical and immunofluorescence images of PIM1 in sections of carotid artery of *ApoE*^-/-^ mice fed a normal diet (ND) and high fat diet (HFD) mice (n = 5). Scale bar of immunohistochemical = 100 μm, Scale bar of immunofluorescence = 50 μm. (I) The PIM1 expression levels analysis based on RNA-seq data from GSE43292. (J) Representative H&E, Masson and immunohistochemical, immunofluorescence images of PIM1 on stable and unstable plaques sections from human carotid artery (n = 10). Scale bar of H&E, Masson and immunohistochemical = 100 μm, Scale bar of immunofluorescence = 500 μm. Data are shown as mean ± SD. *P < 0.05, **P < 0.01, ***P < 0.001.

To identify genes involved in the AS progression through the EndMT process, we reanalyzed previous single-cell sequencing (scRNA-seq) data (GSE155513) 29. We obtained 28816 cells from different plaques in mice fed with a HFD for 0, 8, 16, or 26 weeks after batch correction. Based on key cell markers, we defined 11 cell types including smooth muscle cells (SMC), SMC-derived intermediate stem cells (ICS-SEM), fibrochondrocytes (FC), macrophages 1, 2, 3, minior SMC, fibroblasts1, 2, T cell, and endothelial cell (EC) (Figure 1A-B). Subsequently compared the gene expression levels of endothelial cells from plaques at different stages (Figure 1C). To further identify genes involved in the EndMT process, we analyzed the transcriptome sequencing (RNA-seq) data (GSE118446) 29 of HUVECs-induced EndMT (Figure 1D) and intersected the results with the differentially expressed genes (DEGs) obtained from the scRNA-seq data (Figure 1E). The Venn diagram showed 13 overlapping genes, including FN1, CALD1, COL5A2, COL8A1, EDN1, FBLN5, SDC4, LGMN, LUM, LGALS3, TUBB3, DCN, PIM1. We selected PIM1 for further analysis because, as a signaling factor, it integrates multiple signaling pathways and regulates the expression of various downstream genes. This versatility makes PIM1's role in disease progression more complex and significant compared to other genes with relatively singular functions. Next, we analyzed PIM1 expression levels in four groups of endothelial cells and found that its expression level and proportion increased with the progression of plaques (Figure 1F). We also examined the PIM1 expression of other cells in various stages of atherosclerotic plaques. The results indicate that in smooth muscle cells (SMCs), the number of PIM1-positive cells initially decreased and then increased, while the proportion of PIM1-positive cells consistently rose (Figure S1A). In T cells, the number of PIM1-positive cells continuously increased, but the positive proportion initially decreased before rising again (Figure S1B). In ICS (SEM), the number of PIM1-positive cells showed a decline followed by an increase, with no significant change in proportion (Figure S1C). In fibroblasts, the number of PIM1-positive cells decreased, then increased, and subsequently decreased again, while their proportion steadily rose (Figure S1D). In macrophages, the number of PIM1-positive cells continuously increased, with a sharp rise in the positive proportion during week 8 of plaque formation, followed by a slight decline, but ultimately remained elevated (Figure S1E). To validate the conclusions of the analysis above, we extracted proteins from the thoracic and abdominal aortas of *ApoE*^-/-^ mice subjected to the normal diet or HFD for 16 weeks and assessed PIM1 protein expression. The results indicated that HFD promoted the expression of PIM1 in both the thoracic and abdominal aortas (Figure 1G, Figure S2A). Moreover, immunohistochemical and immunofluorescence staining of carotid artery sections was performed to detect PIM1 in endothelial cells. As expected, the PIM1 protein levels were significantly upregulated in endothelial cells in the carotid atherosclerotic plaques (Figure 1H, Figure S2B). RNA-seq of human carotid atherosclerotic plaques (GSE43292) yielded similar findings, showing higher levels of PIM1 mRNA expression in these plaques (Figure 1I). More importantly, we randomly selected 10 stable and 10 unstable plaques from our own repository of carotid atherosclerotic plaque specimens, obtained through carotid endarterectomy, and performed immunohistochemistry and immunofluorescence staining to detect PIM1 expression levels. The results revealed that PIM1 expression was significantly higher in unstable plaques compared to stable plaques, predominantly localized in the endothelial cells of neo-vessels within the plaques (Figure 1J, Figure S2C). These endothelial cells exhibited distinct mesenchymal characteristics.

These findings indicated that PIM1 expression is markedly elevated in atherosclerotic plaques and is closely associated with EndMT and plaque neovascularization, potentially contributing to plaque instability.

### PIM1 is upregulated in endothelial cells under the conditions of ox-LDL stimulation

**Figure 2:**
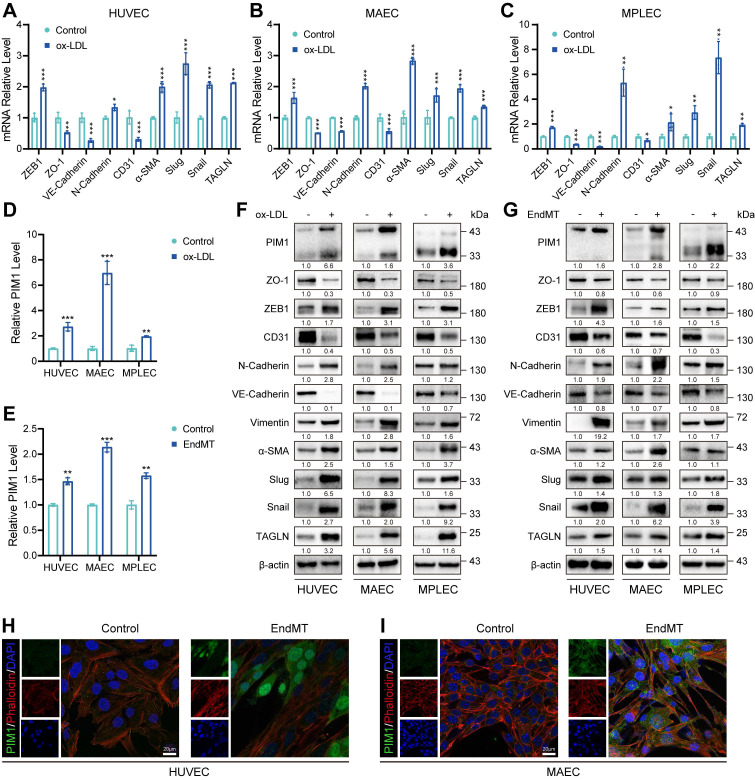
** PIM1 is upregulated in endothelial cells under the conditions of ox-LDL stimulation.** (A-C) qRT-PCR showing the transcript levels of ZEB1, ZO-1, VE-Cadherin. N-Cadherin, CD31, α-SMA, Slug, Snail and TAGLN in HUVEC, MAEC and MPLEC treated with ox-LDL (100 μg/mL, 48 h). (D) qRT-PCR showing the transcript levels of PIM1 in HUVEC, MAEC and MPLEC treated with ox-LDL (100 μg/mL, 48 h). (E) qRT-PCR showing the transcript levels of PIM1 in HUVEC, MAEC and MPLEC treated with H_2_O_2_ (200 μM) and TGF-β (50 ng/mL, 48 h). (F) Representative Western blot images and quantification of PIM1, PIM1, ZO-1, ZEB1, CD31, N-Cadherin, VE-Cadherin, Vimentin, α-SMA, Slug, Snail and TAGLN levels in HUVEC, MAEC and MPLEC treated with ox-LDL (100 μg/mL, 48 h). (G) Representative Western blot images and quantification of PIM1, PIM1, ZO-1, ZEB1, CD31, N-Cadherin, VE-Cadherin, Vimentin, α-SMA, Slug, Snail and TAGLN levels in HUVEC, MAEC and MPLEC treated with H_2_O_2_ (200 μM) and TGF-β (50 ng/mL, 48 h). (H) Representative immunofluorescence images to detect PIM1 expression in 100 μg/mL ox-LDL-stimulated HUVEC. Scale bar = 20 μm. (I) Representative immunofluorescence images to detect PIM1 expression in 100 μg/mL ox-LDL-stimulated MAEC. Scale bar = 20 μm. qRT-PCR Graph is representative of fold change relative to vehicle-treated control cells normalized to 1 (dashed line). Data are shown as mean ± SD. *P < 0.05, **P < 0.01, ***P < 0.001.

Since ox-LDL plays a crucial role in AS, we stimulated HUVECs, (MAECs), and MPLECs with 100 μg/mL ox-LDL. All three cell lines exhibited decreased expression of endothelial markers such as CD31 and increased expression of mesenchymal markers, including ZEB1, Snail, Slug, and α-SMA, under ox-LDL stimulation (Figure 2A-C). This indicates that ox-LDL significantly promotes EndMT. RNA-seq of HUVEC treated with ox-LDL (GSE206927) showed that PIM1 was upregulated compared to the control group (Figure S3A). Similarly, using a previously published method 27, 28 of EndMT induction, HUVEC and MAEC showed results similar to those under ox-LDL stimulation (Figure S3B-C). More importantly, stimulation with either ox-LDL or TGF-β and H_2_O_2_ significantly increased PIM1 expression in endothelial cells (Figure 2D-E). This conclusion was further corroborated by Western blot analysis (Figure 2F-G, Figure S3D-I). Immunofluorescence and cytoskeletal staining demonstrated that EndMT markedly alterd cell morphology and significantly increased PIM1 expression (Figure 2H-I). Furthermore, two distinct isoforms of PIM1 were localized in the cytoplasm and nucleus (Figure 2H-I), with PIM1-L primarily in the cytoplasm and PIM1-S mainly in the nucleus 30.

In conclusion, these data suggested that PIM1 may be involved in endothelial cell function and EndMT.

### PIM1 silence attenuates the process of EndMT

**Figure 3:**
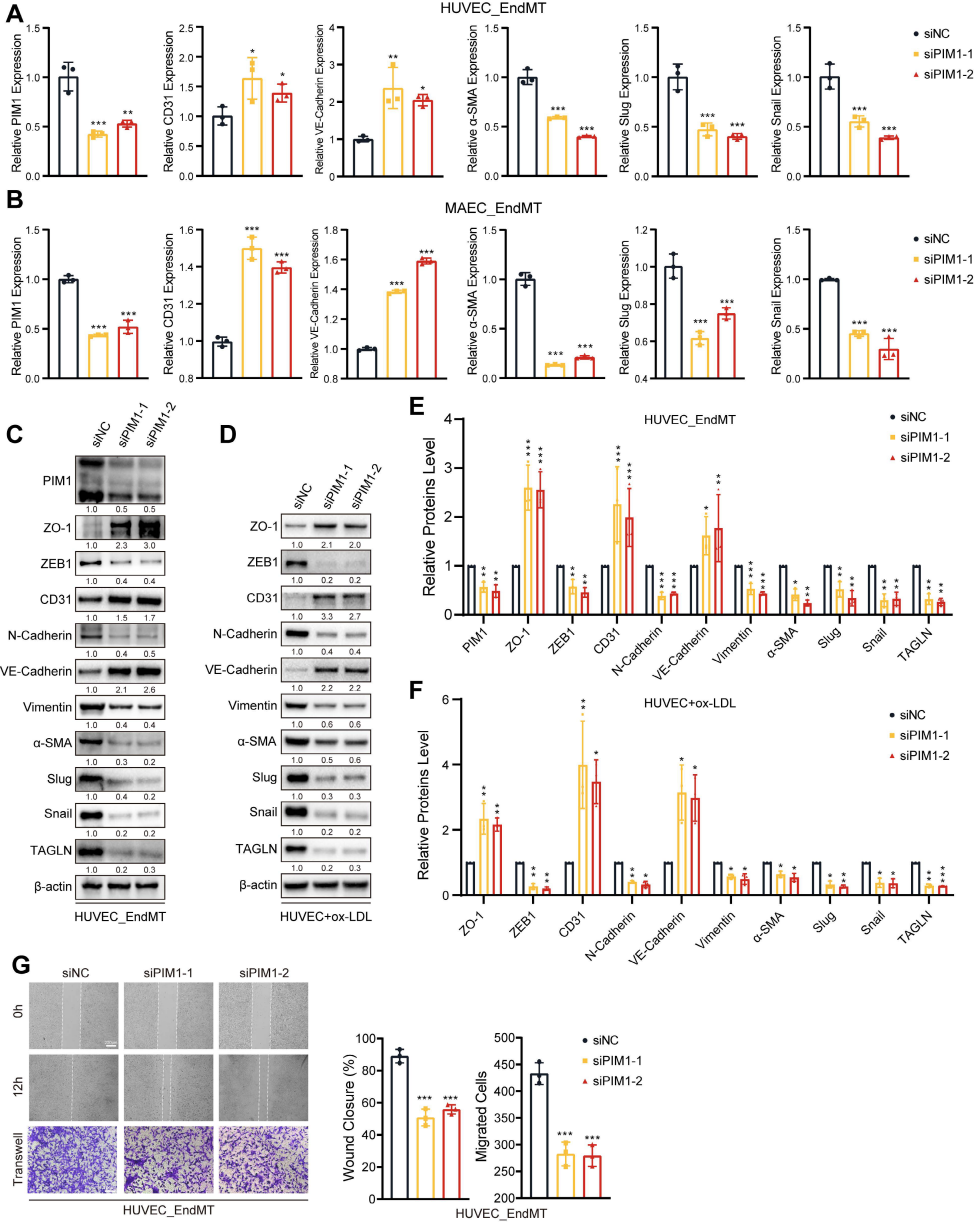
** PIM1 silence attenuates the process of EndMT.** (A) qRT-PCR analysis of PIM1, CD31, VE-Cadherin, α-SMA, Slug and Snail mRNA levels in HUVEC pretreated with siNC or siPIM1-1, siPIM1-2 and stimulated with H_2_O_2_ (200 μM) and TGF-β (50 ng/mL, 48 h). (B) qRT-PCR analysis of PIM1, CD31, VE-Cadherin, α-SMA, Slug and Snail mRNA levels in MAEC pretreated with siNC or siPIM1-1, siPIM1-2 and stimulated with H_2_O_2_ (200 μM) and TGF-β (50 ng/mL, 48 h). (C) Representative Western blot images and quantification of PIM1, ZO-1, ZEB1, CD31, N-Cadherin, VE-Cadherin, Vimentin, α-SMA, Slug, Snail and TAGLN levels in HUVEC pretreated with siNC or siPIM1-1, siPIM1-2 and stimulated with H_2_O_2_ (200 μM) and TGF-β (50 ng/mL, 48 h). (D) Representative Western blot images and quantification of ZO-1, ZEB1, CD31, N-Cadherin, VE-Cadherin, Vimentin, α-SMA, Slug, Snail and TAGLN levels in MAEC pretreated with siNC or siPIM1-1, siPIM1-2 and stimulated with ox-LDL (100 μg/mL, 48 h). (E) Statistical analysis of PIM1, ZO-1, ZEB1, CD31, N-Cadherin, VE-Cadherin, Vimentin, α-SMA, Slug, Snail and TAGLN levels in HUVEC pretreated with siNC or siPIM1-1, siPIM1-2 and stimulated with H_2_O_2_ (200 μM) and TGF-β (50 ng/mL, 48 h). (Figure 3C, n = 3, Normalized to β-actin). (F) Statistical analysis of ZO-1, ZEB1, CD31, N-Cadherin, VE-Cadherin, Vimentin, α-SMA, Slug, Snail and TAGLN levels in MAEC pretreated with siNC or siPIM1-1, siPIM1-2 and stimulated with ox-LDL (100 μg/mL, 48 h). (Figure 3D, n = 3, Normalized to β-actin). (G) Endothelial scratch wound healing assays and Transwell assay were performed, showing that PIM1 silenced attenuated migration of HUVEC induced by H_2_O_2_ (200 μM) and TGF-β (50 ng/mL). Scale bar of wound healing assays = 200 μm. Scale bar of Transwell assay = 100 μm. Data are shown as mean ± SD. *P < 0.05, **P < 0.01, ***P < 0.001.

To investigate the regulatory role of PIM1 in EndMT, we silenced PIM1 expression using siRNA in HUVECs and MAECs and then assessed the expression of endothelial and mesenchymal markers under EndMT induction. Both qRT-PCR and Western blot results indicated that under EndMT induction, knocking down PIM1 could suppress the expression of mesenchymal markers such as α-SMA, Slug, and Snail, while restoring the expression of endothelial markers including CD31 and VE-Cadherin (Figure 3A-C; Figure S4A-D). After knocking down PIM1 in HUVECs, we stimulated the cells with ox-LDL for 48 hours and then extracted proteins. Western blot experiments demonstrated that PIM1 knockdown mitigated ox-LDL-induced EndMT (Figure 3D). PIM447, an inhibitor of PIM1, has the same effect (Figure S5A-D). It has been reported that endothelial cells undergo significant morphological changes and exhibit enhanced migratory ability 1. Therefore, under EndMT-inducing conditions, we assessed the migratory ability of endothelial cells after silencing PIM1 using Wound closure and Transwell assays. The results showed that inhibiting PIM1 expression significantly suppressed the enhanced migratory ability of endothelial cells induced by EndMT (Figure 3G, Figure S6A-C). For atherosclerotic plaques, this would significantly increase the barrier function of vascular endothelium, reducing plaque instability caused by increased endothelial cell migratory ability.

### Endothelial cell-specific PIM1 knockdown reduces EndMT and attenuates atherosclerotic plaque progress

**Figure 4:**
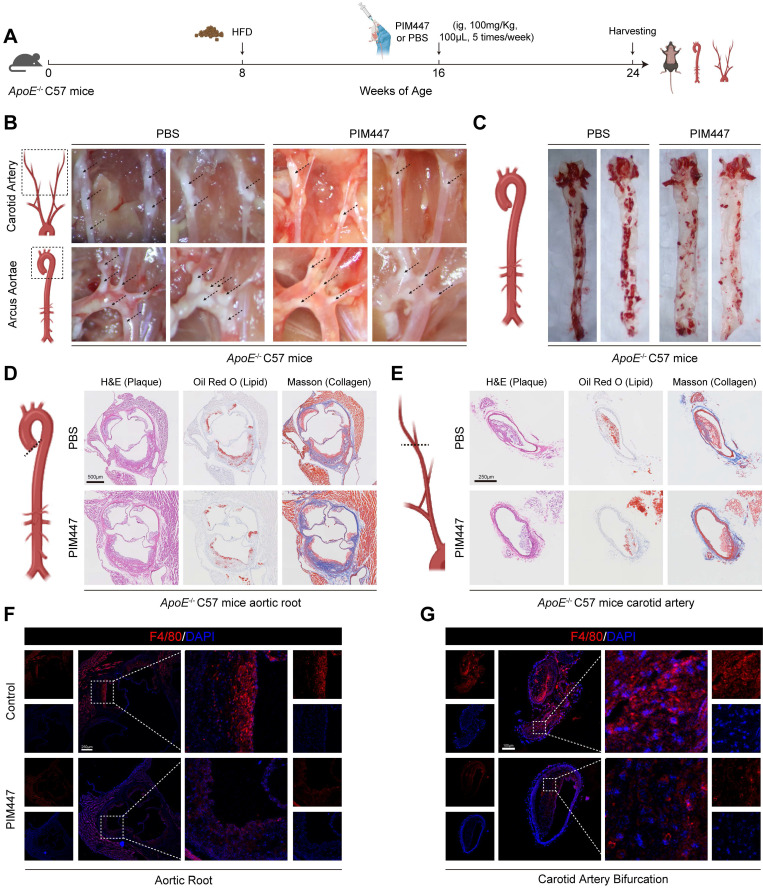
** PIM447 attenuates atherosclerotic plaque progress *in vivo*.** (A) Schematic representation of high-fat diet and pharmacological intervention in C57 *ApoE*^-/-^ mice. (B) Representative photographs of atherosclerotic plaques in the aortic arches and carotid artery in the 2 groups (n = 5). (C) Representative oil red O staining images of the atherosclerotic lesions in the whole aorta in the 2 groups (n = 5). (D) Representative H&E staining images (left), oil red O staining images (middle), Masson staining images (right) of the aortic root in the 2 groups (n = 5). Scale bar = 500 μm. (E) Representative H&E staining images (left), oil red O staining images (middle), Masson staining images (right) of the carotid artery bifurcation in the 2 groups (n = 5). Scale bar = 250 μm. (F) Representative immunofluorescence staining images of F4/80 expression in aortic root sections in the 2 groups (n = 5). Scale bar of aortic root =250 μm. (G) Representative immunofluorescence staining images of F4/80 expression in carotid artery bifurcation sections in the 2 groups (n = 5). Scale bar of carotid artery bifurcation =100 μm. Data are shown as mean ± SD. *P < 0.05, **P < 0.01, ***P < 0.001.

Since PIM1 expression was elevated in the endothelial cells of atherosclerotic plaques, we wanted to determine whether PIM1 knockdown inhibited the process of EndMT and attenuated atherosclerotic plaques progress. Firstly, we conducted a small-scale pilot experiment. *ApoE*^-/-^ mice were fed an HFD to establish an atherosclerotic plaque mouse model. After 8 weeks on the HFD, we initiated PIM447 or PBS intervention (n = 5, every group), which continued for another 8 weeks while maintaining the high-fat diet. The administration of PIM447 and PBS was performed via oral gavage at a dosage of 100 mg/kg, five times a week. Subsequently, we collected the mice's vascular tissues for analysis (Figure 4A). The results showed that PIM447 can prevent the progression of atherosclerotic plaques (Figure 4B-E), reduce the infiltration of macrophages (Figure 4F-G) and endothelial cell inflammation (Figure S7A-B) in plaques. Furthermore, PIM447 can inhibit the process of EndMT (Figure S8A-F).

**Figure 5:**
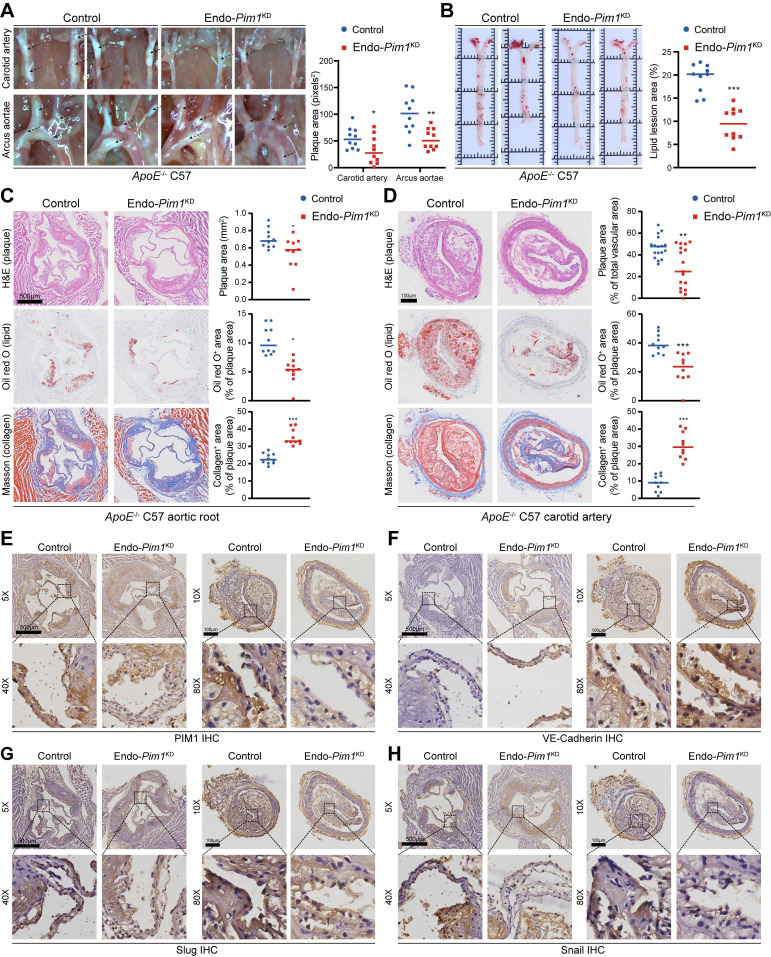
** Endothelial cell-specific PIM1 knockdown reduces EndMT and attenuates atherosclerotic plaque progress.** (A) Representative photographs and plaques area quantification of atherosclerotic plaques in the aortic arches and carotid artery in the 2 groups (n = 10). (B) Representative oil red O staining images and quantification of the atherosclerotic lesions in the whole aorta in the 2 groups (n = 10). (C) Representative H&E staining images (top), oil red O staining images (middle), Masson staining images (bottom) and quantification of the atherosclerotic lesion area, oil red O positive percentage, collagen positive percentage in the aortic root in the 2 groups (n = 10). Scale bar=500 μm. (D) Representative H&E staining images (top), oil red O staining images (middle), Masson staining images (bottom) and quantification of the atherosclerotic lesion area, oil red O positive percentage, collagen positive percentage in the carotid artery bifurcation in the 2 groups (n = 10). Scale bar=100 μm. (E) Representative immunohistochemical staining images of PIM1 protein levels in aortic root and carotid artery bifurcation sections in the 2 groups (n = 10). Scale bar of aortic root =500μm. Scale bar of carotid artery bifurcation =100 μm. (F) Representative immunohistochemical staining images of VE-Cadherin protein levels in aortic root and carotid artery bifurcation sections in the 2 groups (n = 10). Scale bar of aortic root =500 μm. Scale bar of carotid artery bifurcation =100 μm. (G) Representative immunohistochemical staining images of Slug protein levels in aortic root and carotid artery bifurcation sections in the 2 groups (n = 10). Scale bar of aortic root =500 μm. Scale bar of carotid artery bifurcation =100 μm. (H) Representative immunohistochemical staining images of Snail protein levels in aortic root and carotid artery bifurcation sections in the 2 groups (n = 10). Scale bar of aortic root =500 μm. Scale bar of carotid artery bifurcation =100 μm. Data are shown as mean ± SD. *P < 0.05, **P < 0.01, ***P < 0.001.

Endothelial cell-specific *Pim1* knockdown was established by recombinant adeno associated virus serotype 9 (rAAV9) (Figure S9A). After 4 weeks, the PIM1 protein levels in the MPLECs from endothelial cell-specific *Pim1*^KD^ -rAAV9 (Endo-*Pim1*^KD^) and endothelial cell-specific *Control* -rAAV9 (Control) *ApoE*^-/-^ mice (Figure S9B) were detected by Western-blot. The results confirmed the absence of PIM1 expression in endothelial cell from endothelial cell-specific *Pim1*^KD^ -rAAV9 *ApoE*^-/-^mice (Figure S9C). More important, under the EndMT induction, the expression of mesenchymal markers such as α-SMA, Slug, and Snail were downregulated on the MPLEC from Endo-*Pim1*^KD^ mice, while the expression of endothelial markers including CD31 and VE-Cadherin were restored (Figure S9C). Next, endothelial cell-specific *Pim1*^KD^ -rAAV9 *ApoE*^-/-^mice and *Control* -rAAV9 *ApoE*^-/-^ mice were fed an HFD for 16 weeks to develop AS (Figure S9A). *ApoE*^-/-^-Endo-*Pim1*^KD^ mice had fewer atherosclerotic plaques in the aortic arch, carotid artery, and aortic root than the *ApoE*^-/-^-Control mice (Figure 5A-D). In addition, the oil red O staining revealed more minor lipid lesions in the aortic root and carotid artery in *ApoE*^-/-^-Endo-*Pim1*^KD^ mice than in *ApoE*^-/-^-Control mice (Figure 5B-D), Also, Masson staining showed *Pim1* knockdown increased the content of collagen and the ratio of fibrous cap in the aortic root and carotid artery atherosclerosis plaques (Figure 5C-D; Figure S10A-B). PIM1 knockdown also reduced the infiltration of macrophages (Figure S10C) and endothelial cell inflammation (Figure S10D-E) in plaques. We next performed immunohistochemical to assess the expression of PIM1 and EndMT markers in the endothelial cell of aortic root and carotid artery plaques. Compared with plaques from *ApoE*^-/-^-Control mice, plaques from *ApoE*^-/-^-Endo-*Pim1*^KD^ mice showed lower protein levels of PIM1, Slug, Snail, α-SMA, and TAGLN but higher protein levels of VE-Cadherin (Figure 5E-H; Figure S11A-D).

These findings indicated that in the setting of atherosclerosis, the absence of PIM1 in endothelial cells is associated with reduced EndMT, reduced atherosclerotic burden, and a more favorable plaque phenotype.

### PIM1 Promotes the EndMT of Endothelial cell through phosphorylation of NDRG1 at Ser-330

**Figure 6:**
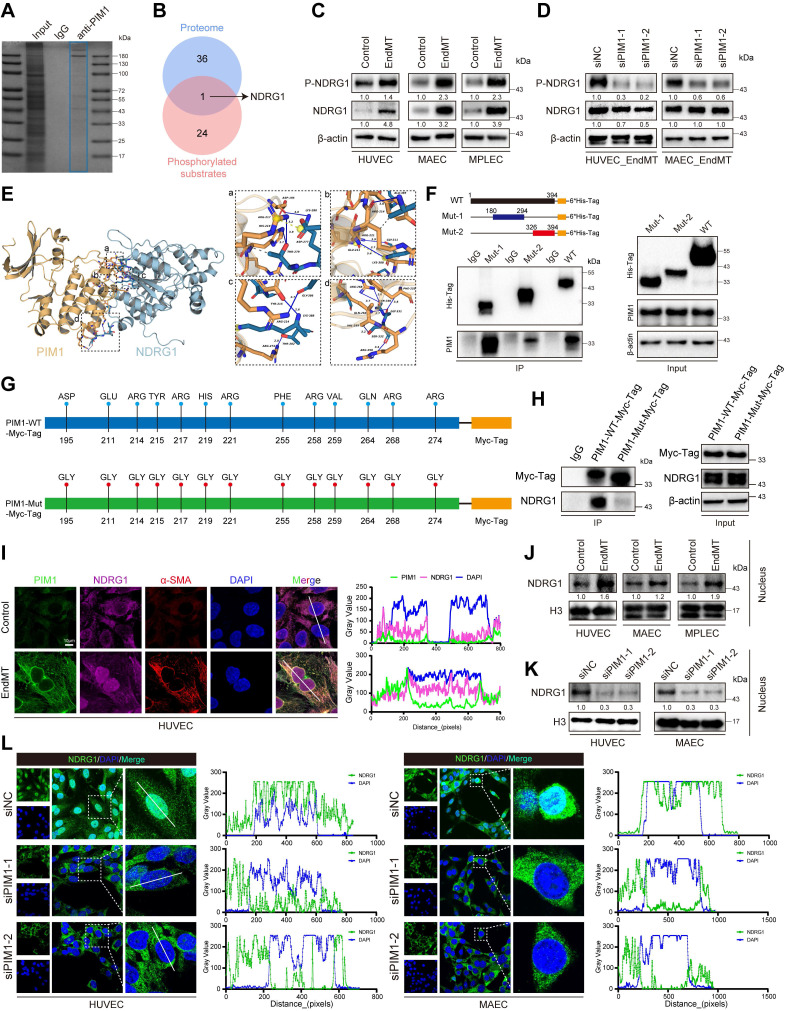
** PIM1 promotes the EndMT of endothelial cell through phosphorylation of NDRG1 at Ser-330.** (A) The proteins of input, IgG and anti-PIM1 were purified and size fractionated on 10% SDS-PAGE. The gel was stained by coomassie brilliant blue staining. (B) Venn diagram showing 1 overlapping protein (NDRG1) between protein mass spectrometry and already reported PIM1 phosphorylated substrates. (C) Representative Western blot images and quantification of P-NDRG1(S330), NDRG1 protein levels in HUVEC, MAEC and MPLEC treated with H_2_O_2_ (200 μM) and TGF-β (50 ng/mL, 48 h). (D) Representative Western blot images and quantification of P-NDRG1(S330), NDRG1 protein levels in HUVEC and MAEC pretreated with siNC or siPIM1-1, siPIM1-2 and stimulated with H_2_O_2_ (200 μM) and TGF-β (50 ng/mL, 48 h). (E) Molecular simulations and protein docking of PIM1 and NDRG1. (F) Schematic diagrams of 6*His-Tagged full-length (WT) NDRG1, and their various deletion mutants (180-294aa, and 326-394aa) (Top). HEK 293T cells were co-transfected with His-Tagged NDRG1 or its deletion mutants or vectors, and whole cell lysates were assessed by immunoprecipitation followed by immunoblotting with anti-His-Tag and anti-PIM1 (bottom). (G) Schematic diagrams of Myc-Tagged wildtype (WT) PIM1, and Myc-Tagged mutant (Mut) PIM1. (H) HEK 293T cells were co-transfected with Myc-Tagged wildtype (WT) PIM1, Myc-Tagged mutant (Mut) PIM1 and whole cell lysates were assessed by immunoprecipitation followed by immunoblotting with anti-Myc-Tag and anti-NDRG1. (I) Fluorescence colocalization and quantification between PIM1 (Green), NDRG1 (Red) and α-SMA in HUVEC. Scale bar = 10 μm. (J) Representative Western blot images and quantification of NDRG1 nuclear protein levels in HUVEC, MAEC and MPLEC treated with H_2_O_2_ (200 μM) and TGF-β (50 ng/mL, 48 h). (K) Representative Western blot images and quantification of NDRG1 Nuclear protein levels in HUVEC and MAEC pretreated with siNC or siPIM1-1, siPIM1-2 and stimulated with H_2_O_2_ (200 μM) and TGF-β (50 ng/mL, 48 h). (L) Fluorescence localization and quantification of NDRG1 (Green) in HUVEC and MAEC pretreated with siNC or siPIM1-1, siPIM1-2 and stimulated with H_2_O_2_ (200 μM) and TGF-β (50 ng/mL, 48 h). Data are shown as mean ± SD. *P < 0.05, **P < 0.01, ***P < 0.001.

We next investigated how PIM1 regulated EndMT. Previously, it was reported that PIM1 is a proto-oncogene, encoding a serine/threonine protein kinase, involved in various biological processes by phosphorylating multiple target substrates 20, 31, 32. Therefore, we first screened its target proteins by immunopurification and MS *in vitro*. To this end, myc-PIM1 was ectopically expressed in 293T cells. The whole-cell extracts were prepared and subjected to affinity purification using Myc-Tag antibody and IgG antibody. The eluted protein complex was then resolved on sodium dodecyl sulfate-polyacrylamide gel electrophoresis, Coomassie Blue stained and subjected to LC-MS/MS analysis (Figure 6A). The identified proteins were listed in Supplementary Table S7. Previous studies have explored the phosphorylation substrates of PIM1 33. Protein kinases promote phosphorylation reactions by binding to the substrates, enhancing the specificity and facilitating conformational changes at the catalytic site, thereby increasing the efficiency of phosphate transfer.

Such binding typically depends on the specific amino acid sequence of the substrate and the binding pocket of the kinase. The affinity and binding kinetics between kinases and substrates are have been shown to be crucial for their function 34-36. Next, by intersecting our mass spectrometry results with phosphorylation substrates, we identified NDRG1 (Figure 6B). We hypothesized that PIM1 promotes EndMT by facilitating the phosphorylation of NDRG1 at the Ser-330 site. Our analysis showed that during EndMT, the phosphorylation level of NDRG1 at Ser-330 was significantly increased (Figure 6C, Figure S12A). However, upon PIM1 knockdown, the phosphorylation of NDRG1 at Ser-330 was inhibited (Figure 6D, Figure S12B), indicating that PIM1 could bind to NDRG1 and promote phosphorylation of the Ser-330 site of NDRG1. We conducted molecular simulations and protein docking to investigate further the binding domains of PIM1 and NDRG1. The binding free energy calculated by the MM/GBSA method using the HawkDock server was -34.31 (kcal/mol). There are 15 hydrogen bonds (within 4.1 Å) and two salt bridges formed between the proteins. As shown in Figure 6E (a), a hydrogen bond (blue solid line) was formed between PIM1's ASP-195 and NDRG1's LYS-280, and hydrogen bonds were formed between PIM1's ARG-217 and HIS-219 with NDRG1's THR-279, with a salt bridge (yellow dashed line) also formed between PIM1's ARG-217 and NDRG1's ASP-277. In Figure 6E (b), a hydrogen bond was formed between NDRG1's ALA-385 and PIM1's ARG-214, another between PIM1's ARG-221 and NDRG1's GLY-311, along with a hydrogen bond and salt bridge between NDRG1's LYS-388 and PIM1's GLU-211. In Figure 6E (c), a hydrogen bond is formed between NDRG1's GLY-386 and PIM1's ARG-214, another between PIM1's TYR-215 and NDRG1's LYS-388, and yet another between NDRG1's THR-282 and PIM1's ARG-274. Finally, in Figure 6E (d), two hydrogen bonds were formed between PIM1's ARG-268 and NDRG1's SER-330, and hydrogen bonds were formed between NDRG1's GLY-331 and PIM1's PHE-255 and GLN-264, as well as between NDRG1's SER-332 and PIM1's VAL-259 and ARG-258. Figure 6E also shows the presence of multiple hydrophobic interactions. Furthermore, as displayed in Figure S12C, in HUVECs, immunoprecipitation of PIM1 and NDRG1 resulted in co-immunoprecipitation of NDRG1 and PIM1, respectively, but the anti-IgG antibody did not immunoprecipitate PIM1 or NDRG1. We therefore performed *in vitro* SPR assays, which demonstrated considerable affinities and direct binding between PIM1 and NDRG1 (Figure S12D). Based on the protein docking results, we designed two truncated mutants of NDRG1 spanning amino acids 180-294 and 326-394 and overexpressed them with the wild-type NDRG1-His-Tag, in 293T cells. Using an anti-His-Tag antibody for immunoprecipitation, we found that both truncated mutants could bind to PIM1 (Figure 6F). Given that PIM1 has only one structural domain, we mutated the amino acids that form hydrogen bonds with NDRG1 according to the docking results (Figure 6G). Subsequent Co-IP experiments confirmed that the binding affinity between PIM1 and NDRG1 decreased after mutation (Figure 6H). This further validated our molecular docking results. Confocal immunofluorescent staining demonstrated strong localization of PIM1 and NDRG1 in HUVECs and MAECs (Figure 6I, Figure S12E). Unexpectedly, under EndMT-inducing conditions, NDRG1 appeared to have increased expression in the nucleus (Figure 6I, Figure S12E). To further validate this phenomenon, we performed nuclear-cytoplasmic fractionation followed by Western blot analysis. The results indicated that EndMT induction promoted the nuclear translocation of NDRG1 in endothelial cells (Figure 6J, Figure S12F), while PIM1 knockdown inhibited this process (Figure 6K-L, Figure S12G). Previous studies have also suggested that phosphorylation of NDRG1 at the Ser-330 site may facilitate its nuclear translocation 37, which is consistent with our findings.

### NDRG1 is required for PIM1-Induced EndMT

**Figure 7:**
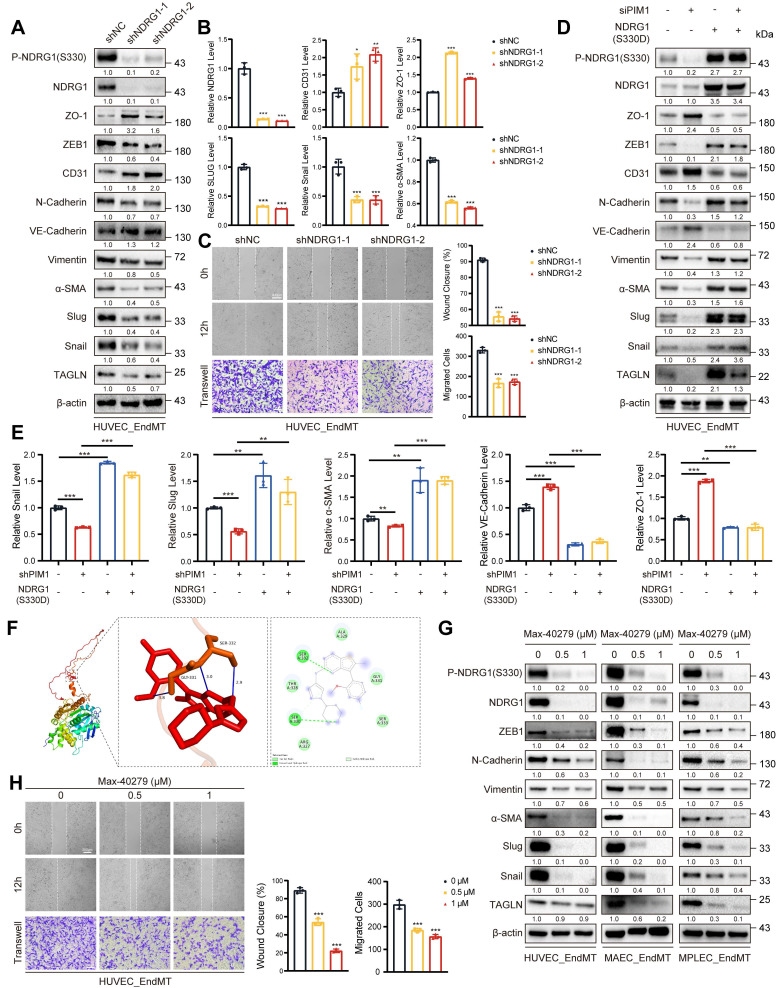
** NDRG1 is Required for PIM1-Induced EndMT.** (A) Representative Western blot images and quantification of P-NDRG1(S330), NDRG1, ZO-1, ZEB1, CD31, N-Cadherin, VE-Cadherin, Vimentin, α-SMA, Slug, Snail and TAGLN levels in HUVEC pretreated with shNC or shNDRG1-1, shNDRG1-2 and stimulated with H_2_O_2_ (200 μM) and TGF-β (50 ng/mL, 48 h). (B) qRT-PCR analysis of NDRG1, CD31, ZO-1, Slug, Snail and α-SMA mRNA levels in HUVEC pretreated with shNC or shNDRG1-1, shNDRG1-2 and stimulated with H_2_O_2_ (200 μM) and TGF-β (50 ng/mL, 48 h). (C) Endothelial scratch wound healing assays and Transwell assay were performed, showing that NDRG1 silenced attenuated migration of HUVEC induced by H_2_O_2_ (200 μM) and TGF-β (50 ng/mL). Scale bar of wound healing assays = 400 μm. Scale bar of Transwell assay = 100 μm. (D) Representative Western blot images and quantification of P-NDRG1(S330), NDRG1, ZO-1, ZEB1, CD31, N-Cadherin, VE-Cadherin, Vimentin, α-SMA, Slug, Snail and TAGLN levels in HUVEC pretreated as indicated and stimulated with H_2_O_2_ (200 μM) and TGF-β (50 ng/mL, 48 h). (E) qRT-PCR analysis of Snail, Slug, α-SMA, VE-Cadherin and ZO-1 mRNA levels in HUVEC pretreated as indicated and stimulated with H_2_O_2_ (200 μM) and TGF-β (50 ng/mL, 48 h). (F) The docking prediction of NDRG1 with Max-40279. (G) Representative Western blot images and quantification of P-NDRG1(S330), NDRG1, ZEB1, N-Cadherin, Vimentin, α-SMA, Slug, Snail and TAGLN levels in HUVEC, MAEC and MPLEC stimulated with H_2_O_2_ (200 μM) and TGF-β (50 ng/mL, 48 h), and treated with Max-40279 (0.5 μM or 1 μM, 48 h). (H) Endothelial scratch wound healing assays and Transwell assay were performed, showing that Max-40279 attenuated migration of HUVEC induced by H_2_O_2_ (200 μM) and TGF-β (50 ng/mL, 48 h). Scale bar of wound healing assays = 400 μm. Scale bar of Transwell assay = 100μm. Data are shown as mean ± SD. *P < 0.05, **P < 0.01, ***P < 0.001.

We have demonstrated that PIM1 promoted the NDRG1 phosphorylation at the Ser-330 site, but the regulatory role of NDRG1 in EndMT remained unknown. Therefore, we constructed NDRG1-knockdown in HUVECs using lentivirus and assessed EndMT expression levels under EndMT-inducing conditions. The results showed that NDRG1 knockdown inhibited the expression of mesenchymal markers such as ZEB1, α-SMA, Slug, Snail, and TAGLN, while restoring the expression of endothelial markers like CD31 and VE-Cadherin (Figure 7A-B, Figure S13A). NDRG1 knockdown also reduced the migratory ability of HUVECs (Figure 7C). Furthermore, after knocking down PIM1, we constructed a phospho-mimetic NDRG1 mutant plasmid, NDRG1(S330D), for rescue experiments. The results showed that overexpressing NDRG1(S330D) completely abolished the inhibitory effect of PIM1 knockdown or PIM447 on EndMT and further promoted EndMT (Figure 7D-E, Figure S13B-C).

Small-molecule drugs have significant advantages in inhibiting protein-protein interactions and blocking phosphorylation and other protein modification sites. Therefore, we downloaded 11,586 small-molecule drugs from DrugBank (https://go.drugbank.com/) and performed high-throughput docking with the Ser-330 site of NDRG1 using AutoDock Vina software. We selected the top five small molecule drugs with the lowest binding energies as potential inhibitors for visualization analysis and validation (Table S8, Figure 7F).

Furthermore, we performed *in vitro* SPR assays, which demonstrating considerable affinities and direct binding between Max-40279 and NDRG1 (Figure S14A). Finally, we demonstrated that Max-40279 significantly inhibits the phosphorylation of NDRG1 at Ser-330 (Figure 7G, Figure S14B-D). Additionally, the presence of Max-40279 also reduced the total protein level of NDRG1. We speculate that Max-40279 may promote the degradation of NDRG1 upon binding, though we did not investigate this further. Western blot results showed that under EndMT-inducing conditions, the addition of Max-40279 in HUVECs, MAECs, and MPLECs significantly inhibited the expression of mesenchymal markers such as ZEB1, α-SMA, Slug, Snail, and TAGLN (Figure 7G, Figure S14B-D), as well as the migratory capacity of endothelial cells (Figure 7H, Figure S14E, F).

Thus, we have shown that NDRG1 mediates PIM1-induced EndMT, while Max-40279 can impede EndMT by inhibiting NDRG1 phosphorylation at Ser-330.

### NDRG1 and PTBP1 collaborate to promote EndMT

**Figure 8:**
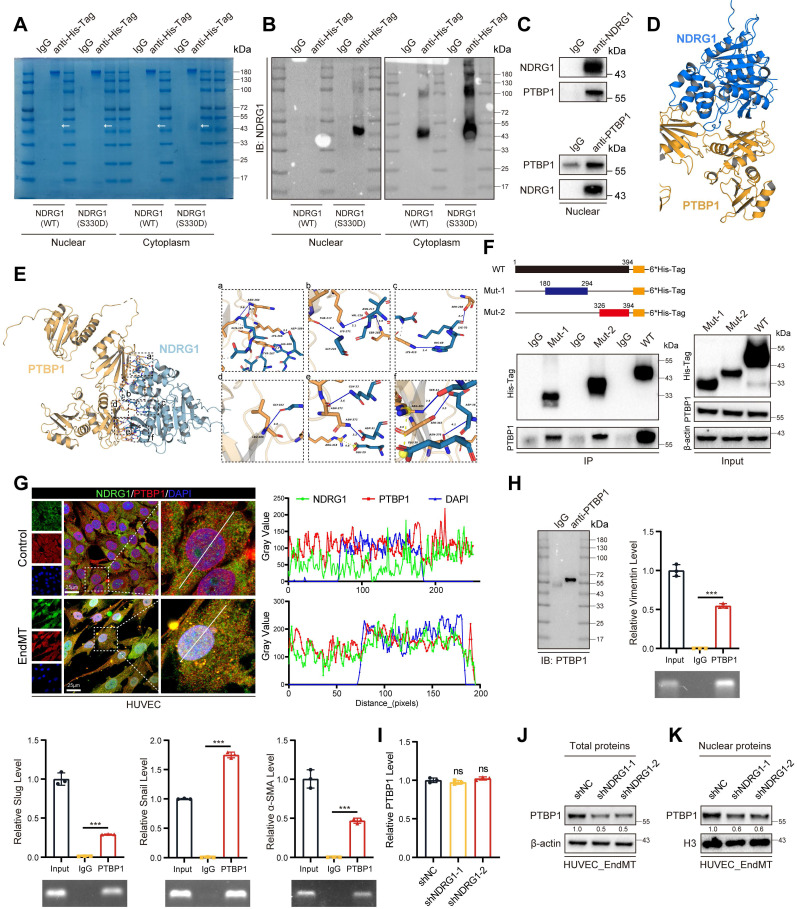
** NDRG1 and PTBP1 collaborate to promote EndMT.** (A) The Nuclear and cytoplasm proteins of input, IgG and anti-His-Tag were purified and size fractionated on 10% SDS-PAGE. The gel was stained by coomassie brilliant blue staining. (B) The content of NDRG1 was analyzed by NDRG1 antibody. (C) The Co-IP experiment detecting the interaction between NDRG1 and PTBP1 in nucleus from HUVEC treated with H_2_O_2_ (200 μM) and TGF-β (50 ng/mL, 48 h). (D, E) Molecular simulations and protein docking of NDRG1 and PTBP1. (F) Schematic diagrams of 6*His-Tagged full-length (WT) NDRG1, and their various deletion mutants (180-294aa, and 326-394aa) (Top). HEK 293T cells were co-transfected with His-Tagged NDRG1 or its deletion mutants or vectors, and whole cell lysates were assessed by immunoprecipitation followed by immunoblotting with anti-His-Tag and anti-PTBP1 (bottom). (G) Fluorescence colocalization and quantification between NDRG1 (Green) and PTBP1 (Red) in HUVEC. Scale bar = 25 μm. (H) Immunopurification of PTBP1/RNA complexes or control experiments (IgG) from HUVEC cell extracts. Immunopurification was controlled by PTBP1 Western blot analysis as indicated in IgG and IP samples (left). qRT-PCR and agarose gel electrophoresis after reverse transcription and PCR detection the different mRNA level (right). (I) qRT-PCR analysis of PTBP1 mRNA levels in HUVEC pretreated with shNC or shNDRG1-1, shNDRG1-2 and stimulated with H_2_O_2_ (200 μM) and TGF-β (50 ng/mL, 48 h). (J) Representative Western blot images and quantification of total PTBP1 protein levels in HUVEC pretreated with shNC or shNDRG1-1, shNDRG1-2 and stimulated with H_2_O_2_ (200 μM) and TGF-β (50 ng/mL, 48 h). (K) Representative Western blot images and quantification of nuclear PTBP1 protein levels in HUVEC pretreated with shNC or shNDRG1-1, shNDRG1-2 and stimulated with H_2_O_2_ (200 μM) and TGF-β (50 ng/mL, 48 h). Data are shown as mean ± SD. *P < 0.05, **P < 0.01, ***P < 0.001.

Although phosphorylation of the Ser-330 site of NDRG1 has been shown to facilitate its nuclear translocation, the underlying mechanisms promoting EndMT within the nucleus remain unclear. To elucidate this, we overexpressed NDRG1 (S330D) in HUVECs and performed proteomic analysis of nuclear proteins (Figure 8A-B). The identified proteins their enrichment levels and *p*-values were listed in supplementary Table 9. Our findings revealed that NDRG1 interacted with PTBP1 in the nucleus. Several studies have demonstrated that PTBP1 could promote EMT 38-40, and advance AS progression by promoting endothelial cell inflammation 41. These findings are consistent with our study. Immunoprecipitation of NDRG1 in HUVECs led to co-immunoprecipitation of PTBP1. Similarly, immunoprecipitation of PTBP1 resulted in co-immunoprecipitation of NDRG1, whereas the anti-IgG antibody did not immunoprecipitate PIM1 or NDRG1 (Figure 8C). Next, molecular simulations and protein docking were performed to investigate further the binding domains of NDRG1 and PTBP1 (Figure 8D). Using the HawkDock server, the calculated binding free energy was -66.29 kcal/mol, with 14 hydrogen bonds (within 4.1 Å) and one salt bridge forming between the proteins. As shown in Figure 8E (a), a hydrogen bond is formed between ASN-264 of PTBP1 and GLN-185 of NDRG1 (blue solid line), between LYS-266 of PTBP1 and ASP-189 of NDRG1, and between HIS-209 of NDRG1 and TYR-267 of PTBP1. In Figure 8E (b), a hydrogen bond is formed between GLY-222 of NDRG1 and THR-217 of PTBP1, between LYS-271 of PTBP1 and VAL-216 of NDRG1, and between ASN-217 of NDRG1 and SER-285 of PTBP1. Figure 8E (c) shows hydrogen bonds forming between HIS-69 of NDRG1 and LYS-410 of PTBP1, and between LYS-70 of NDRG1 and SER-288 of PTBP1. In Figure 8E (d), a hydrogen bond is formed between LEU-408 of PTBP1 and GLY-102 of NDRG1. As shown in Figure 8E (e), a hydrogen bond between GLN-33 of NDRG1 and ASN-372 of PTBP1, with ASN-372 forming an additional hydrogen bond with ASP-31 of NDRG1, and a salt bridge between GLU-29 of NDRG1 and ARG-418 of PTBP1. Figure 8E (f) shows a hydrogen bond forms between ARG-405 of PTBP1 and SER-43 of NDRG1, while a salt bridge forms between ARG-405 of PTBP1 and GLU-38 of NDRG1 (yellow dashed line). Additionally, ASP-36 of NDRG1 forms hydrogen bonds with both SER-342 and ASN-376 of PTBP1. Furthermore, multiple hydrophobic interactions (grey dashed lines) were observed (Figure 8D-E). We also performed *in vitro* SPR assays, demonstrating considerable affinities and direct binding between NDRG1 and PTBP1 (Figure S15A). We then overexpressed the wild-type NDRG1-His-Tag, the mutant NDRG1 (180-294aa)-His-Tag, and the mutant NDRG1 (326-394aa)-His-Tag that were previously constructed in 293T cells. Using an anti-His-Tag antibody for immunoprecipitation, we found that both truncated mutants could bind to PTBP1 (Figure 8F). Additionally, confocal immunofluorescent staining demonstrated a predominantly localization of NDRG1 and PTBP1 in HUVECs and MAECs, particularly under conditions that induce EndMT, NDRG1 and PTBP1 exhibit a stronger colocalization predominantly within the nucleus. (Figure 8G, Figure S15B). It is well known that PTBP1 is an RNA-binding protein. We subsequently performed an RNA Binding Protein Immunoprecipitation (RIP) assay to detect the binding of PTBP1 to the mRNAs of Vimentin, Slug, Snail, α-SMA, N-Cadherin, TAGLN, and ZEB1. The results indicated that PTBP1 can bind to all these mRNAs (Figure 8H, Figure S16A). Additionally, we used protein-RNA docking methods to further confirm these interactions (Figure S16B-H). These findings indicate that the process by which phosphorylated NDRG1 promoted EndMT was dependent on the RNA-binding function of PTBP1. Further investigation revealed that knocking down NDRG1 results in a decrease in PTBP1 levels within the cell, particularly in the nucleus, without affecting PTBP1 mRNA levels. (Figure 8I-K, Figure S16I) This suggests that NDRG1 may enhance the stability of PTBP1, protecting it from degradation.

Together, these data strongly supported the notion that PIM1 expression was significantly upregulated under ox-LDL stimulation. PIM1 promoted the phosphorylation of NDRG1 at the Ser330 site, facilitating NDRG1's translocation into the nucleus. Once inside the nucleus, NDRG1, through the RNA-binding function of PTBP1, promotes EndMT, leading to decreased stability of atherosclerotic plaques and accelerated progression of atherosclerosis. Therefore, targeting the PIM1/NDRG1/PTBP1 axis may represent a novel strategy for treating AS.

## Discussion

AS is the most common cause of cardiovascular and cerebrovascular diseases. The rupture of carotid atherosclerotic plaques can lead to stroke, severely impacting the quality of life. It is a chronic process involving cellular phenotypic transformation, metabolic alterations, and inflammatory events 42, 43. EndMT is believed to play a crucial role in the initiation and progression of AS 28, 44. EndMT is a process whereby an endothelial cell undergoes a series of molecular events that lead to a change in phenotype toward a mesenchymal cell (e.g. myofibroblast, smooth muscle cell)^45^. There are no universally agreed-upon criteria for defining EndMT at the molecular level. However, it is primarily characterized by the downregulation of endothelial markers such as CD31 and VE-Cadherin, and the upregulation of mesenchymal markers such as Vimentin, ZEB1, N-Cadherin, Slug, Snail, α-SMA, and TAGLN. Many characteristics of atherosclerotic plaques, including hypoxia, disturbed flow, oxidative stress, and the TGF-β signaling pathway can activate EndMT 28. As the innermost layer of blood vessels, endothelial cells play crucial roles in maintaining selective permeability barriers, coordinating leukocyte transport, preventing thrombus formation, and regulating vascular tone. During EndMT, endothelial cells undergo functional impairment, lose their polarity, and reshape endothelial cell-cell connections, resulting in the migration of monocytes and macrophages into the vessel wall, increased retention of low-density lipoprotein, and the initiation of atherosclerotic plaque formation. The vicious cycle between EndMT and AS can be explained by the fact that endothelial cells experiencing EndMT establish a positive feedback loop, which further exacerbates endothelial dysfunction and leads to the onset and progression of atherosclerosis. Indeed, besides atherosclerosis, EndMT plays a significant role in various other cardiovascular and cerebrovascular-related diseases, including valvular disease, fibroelastosis, vein graft remodeling, cardiac fibrosis, and pulmonary hypertension 46.

Our results indicated that prolonged exposure of endothelial cells to a high-lipid environment, induced EndMT. This process results in the loss of some endothelial cell characteristics, enhanced migratory ability, and weakened barrier function, thereby facilitating the infiltration of macrophages and other cells into the subendothelial space 47. Our data also showed that stimulation by ox-LDL significantly upregulates the expression of PIM1 in endothelial cells. PIM1, a serine/threonine kinase, increases the phosphorylation of NDRG1 at the Ser330 site. Phosphorylated NDRG1 undergoes subcellular localization changes, altering its mode of action within the cell. Specifically, phosphorylated NDRG1 translocates from the cytoplasm to the nucleus, where it interacts with PTBP1. This interaction inhibits the degradation of PTBP1, allowing PTBP1 to bind to the RNAs of Vimentin, TAGLN, α-SMA, Slug, Snail, and ZEB1, thereby promoting EndMT. However, the mechanisms underlying EndMT require further investigation.

Our study also revealed increased PIM1 expression with the progression of AS, with higher expression in unstable plaques. Single-cell sequencing data that the increase in PIM1 occurs mainly in endothelial cells, macrophages and SMCs. Immunofluorescence staining confirmed the upregulation of PIM1 mainly occurs in the endothelium of neo-vessels within unstable plaques and macrophages surrounding these neo-vessels, while it is scarce in SMCs. This difference may be attributed to the fact that single-cell sequencing is based on transcriptome data, whereas immunofluorescence staining is based on protein expression. These data indicated that the expression and localization of PIM1 not only differ at the transcriptional level but also suggest that post-translational modifications likely play a crucial role in PIM1 expression. In addition to our study, others have also demonstrated that PIM1 plays an important role in endothelial regulation. For example, PIM1 can directly phosphorylates and stabilizes HIF-1α to drive angiogenesis in solid tumors^48^. Furthermore, PIM1 promotes angiogenesis through phosphorylation of endothelial nitric oxide synthase at Ser-633 32. Together with our findings, these studies reinforce the fact that PIM1 is of critical importance for governing endothelial function and pathobiology. As for why PIM1 is also highly expressed in macrophages surrounding neovessels, our study did not delve into this aspect, which is a limitation of our research. Based on existing studies, we speculate that PIM1 may promote the inflammatory response of macrophages 49-51. Inflammatory macrophages tend to accumulate around neovessels, leading to the instability of atherosclerotic plaques and adverse outcomes. In light of this finding, we have begun to investigate in depth the role and mechanism of PIM1 in macrophages.

Although our study demonstrates that PIM1 promotes the phosphorylation of NDRG1, leading to its nuclear translocation and the subsequent progression of EndMT and atherosclerosis, it is important to note that other mechanisms by which NDRG1 promotes atherosclerosis progression still exist. For example, Zhang et.al identified NDRG1 as a critical mediator implicated in regulating endothelial inflammation and vascular remodeling through regulating Nur77/NF-κB and AP1 transcriptional pathways 52. In addition to promoting AS, NDRG1 also contributes to the progression of other diseases through the mechanisms of EndMT or epithelial-to-mesenchymal transition (EMT). NDRG1 facilitates the progression of bladder cancer 53, liver cancer 54, ovarian cancer 55, prostate cancer 56, and esophageal squamous cell carcinoma 57 via EMT. We identified numerous small-molecule drugs that can bind to the Ser330 site on NDRG1 through molecular docking. These molecules could potentially act as inhibitors or activators. Based on their binding free energies, we selected the top 5 small molecule drugs for validation. We purchased Max-40279, Naftazone, and Stenoparib from MCE to validate their effects on the phosphorylation level of NDRG1. Naftazone and Stenoparib did not affect NDRG1 phosphorylation at the Ser330 site, while Max-40279 significantly reduced the phosphorylation level of NDRG1. Surprisingly, Max-40279 also significantly reduced the protein level of NDRG1. This indicates that regardless of the mechanism by which NDRG1 promotes the progression of atherosclerosis, Max-40279 could be a potential therapeutic option for atherosclerosis by inhibiting NDRG1. Max-40279 is a dual inhibitor of FLT3 kinase and FGFR kinase. It has the potential for researching acute myeloid leukemia (AML) (Patent WO2021180032A1). Due to the lack of reference for dosages in mice, this study did not verify the therapeutic effect of Max-40279 in an AS mouse model, which is a limitation of this study. We plan to explore the dosage and efficacy through the use of nanovesicles targeted to plaques in future studies, aiming to provide new strategies for the treatment of AS.

In conclusion, our study demonstrates a unique role for PIM1 in vascular pathology by promoting EndMT, which is associated with increased AS and an unstable plaque phenotype. Furthermore, we have shown that PIM1 inhibition effectively attenuates EndMT *in vitro* and *in vivo*. The mechanism by which PIM1 promotes AS is mainly through the PIM1/NDRG1(Ser330)/PTBP1/EndMT pathway. Targeting of PIM1 or NDRG1 may be beneficial for plaque stabilization or slowing the progression of atherosclerotic disease.

## Supplementary Material

Supplementary figures and tables.
