# Supplementary material for: PIM1 instigates endothelial-to-mesenchymal transition to aggravate atherosclerosis
Source: Theranostics. 2025 Jan 1;15(2):745–65. doi: 10.7150/thno.102597 (PMC11671384; doi:10.7150/thno.102597)
Supplement: Supplementary file 1 — Supplementary figures and tables. [file thnov15p0745s1.pdf]

Supplementary Information

PIM1 instigates endothelial-to-mesenchymal transition to aggravate atherosclerosis

Author name and affiliations:

Zhiwei Xue[1, 2*], Mengtao Han[1, 2*], Tao Sun[1, 2*], Yanzhao Wu[1, 2], Wenchen Xing[1, 2], Feiyu Mu[1, 2], Zhihan Zhang[1, 2], Junzhi Liu[1, 2], Xiangjun Liang[1, 2], Lu Ling[1, 2], Jian Wang[1, 2, 5], Jiwei Wang[1, 2], Xingang Li[1, 2, 3, 4], Bin Huang[1, 2, 3, 4], Donghai Wang[1, 2, 3]

1. Department of Neurosurgery, Qilu Hospital, Cheeloo College of Medicine and Institute of Brain and Brain-Inspired Science, Shandong University.

2. Shandong Key Laboratory of Brain Health and Function Remodeling, Jinan 250012, China.

3. Department of Neurosurgery, Qilu Hospital of Shandong University Dezhou Hospital, Dezhou, China

4. Jinan Microecological Biomedicine Shandong Laboratory and Shandong Key Laboratory of Brain Function Remodeling, Jinan, China

5. Department of Biomedicine, University of Bergen, Jonas Lies Vei 91, 5009, Bergen, Norway.

* Zhiwei Xue, Mengtao Han and Tao Sun contributed equally to this work

#Corresponding Author:

Donghai Wang, Ph.D. and M.D. (drwangdonghai@sdu.edu.cn)

Bin Huang, Ph.D. (hb@sdu.edu.cn)

Xingang Li, Ph.D and M.D. (lixg@sdu.edu.cn)

This supplementary information contains:

- 44 Pages
- Supplementary Figures (16 Figures, S1 to S16)
- Supplementary Tables (9 Tables, S1 to S9)

Supplementary Figures

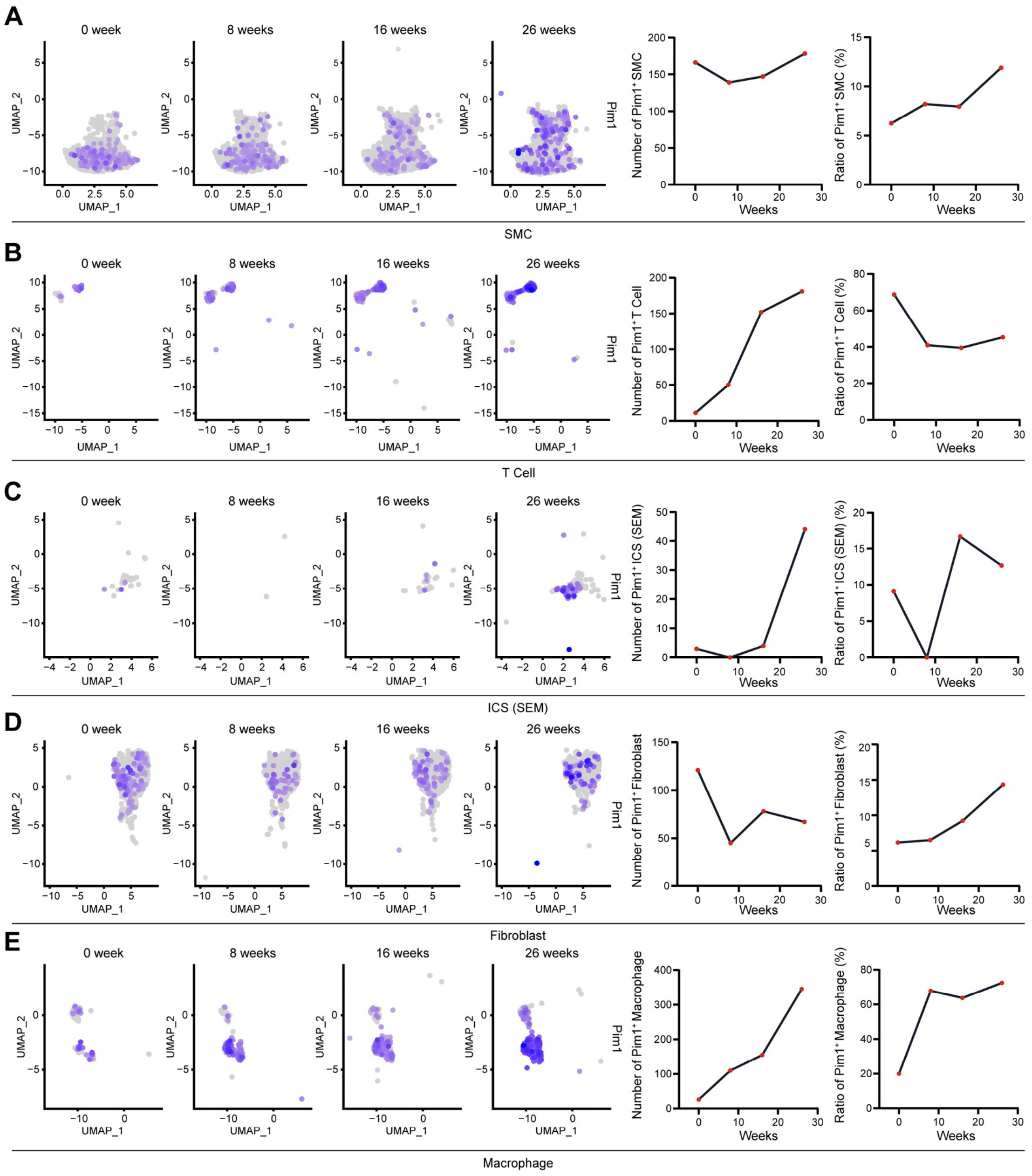

Figure S1. The expression level of PIM1 in different cells of atherosclerotic plaques at different time points.

(A) The expression level of PIM1 in SMC of atherosclerotic plaques at different time points.

(B) The expression level of PIM1 in T cell of atherosclerotic plaques at different time

points.

(C) The expression level of PIM1 in ICS(SEM) of atherosclerotic plaques at different time points.

(D) The expression level of PIM1 in Fibroblast of atherosclerotic plaques at different time points.

(E) The expression level of PIM1 in Macrophage of atherosclerotic plaques at different time points.

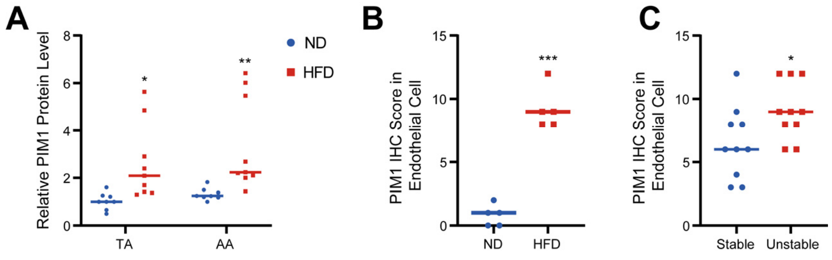

Figure S2. Statistical analysis of PIM1 expression level.

(A) Statistical analysis of PIM1 levels in arcus aortae and thoracic aorta from normal diet (ND) and high fat diet (HFD) mice (Figure 1G, CD group n = 8, HFD group n = 9, Normalized to β-actin).

(B) Statistical analysis of PIM1 IHC Score in endothelial cells of carotid artery of *ApoE*$^{-/-}$ mice fed a normal diet (ND) and high fat diet (HFD) mice (n = 5).

(C) Statistical analysis of PIM1 IHC Score in endothelial cells of stable and unstable plaques sections from human carotid artery (n = 10).

Data are shown as mean ± SD. **P* < 0.05, ***P* < 0.01, ****P* < 0.001.

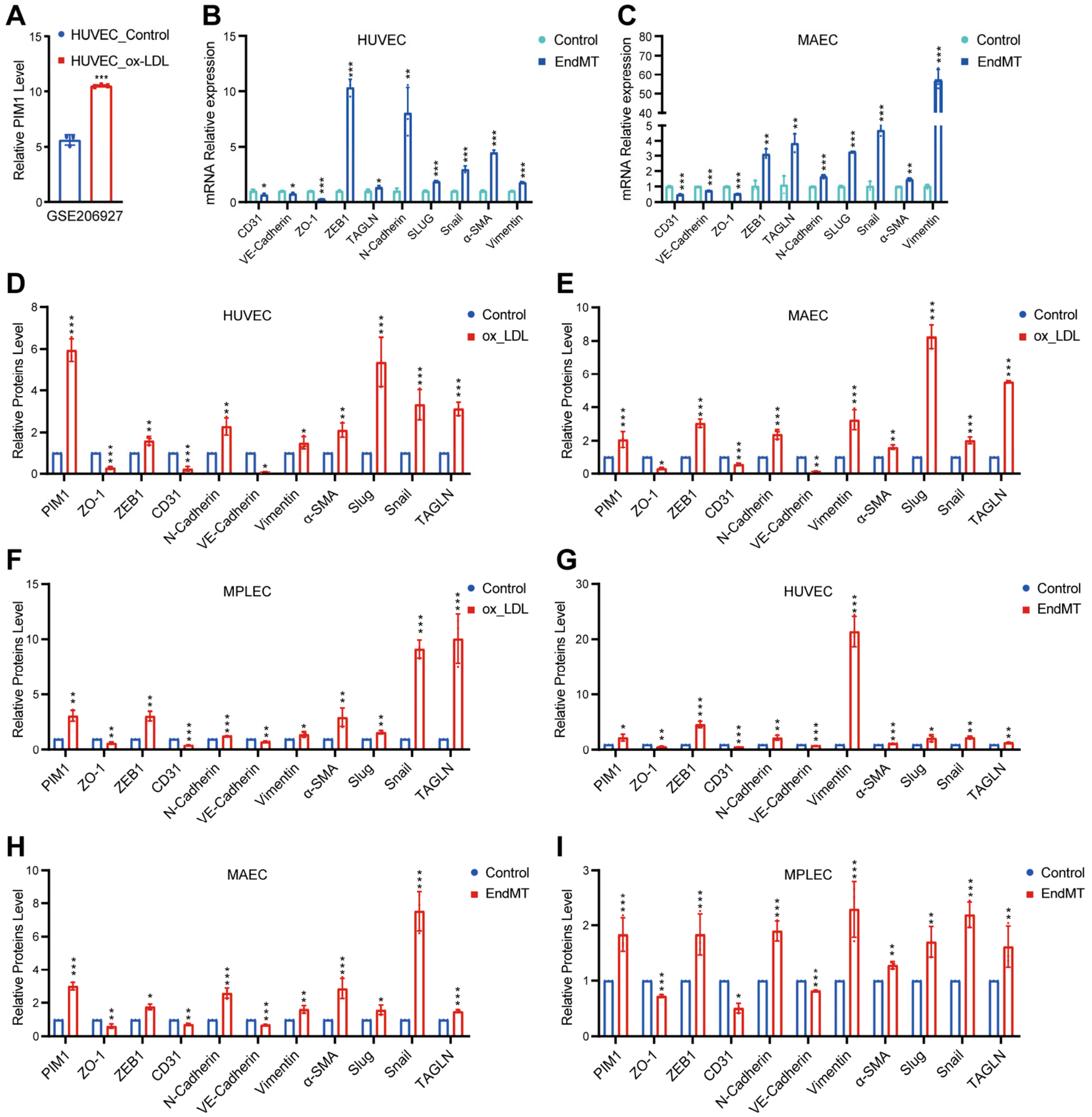

Figure S3. PIM1 is Upregulated in Endothelial cells under the Conditions of ox-LDL Stimulation.

(A) The PIM1 expression levels analysis based on RNA-seq data from GSE206927.

(B) qRT-PCR showing the transcript levels of CD31, VE-Cadherin, ZO-1, ZEB1, TAGLN, N-Cadherin, SLUG, Snail, α-SMA, and vimentin in HUVEC treated with H_2O_2 (200 μM) and TGF-β (50 ng/mL, 48 h).

(C) qRT-PCR showing the transcript levels of CD31, VE-Cadherin, ZO-1, ZEB1, TAGLN, N-Cadherin, SLUG, Snail, α-SMA, and vimentin in MAEC treated with H_2O_2 (200 μM) and TGF-β (50 ng/mL, 48 h).

(D-F) Statistical analysis of PIM1, ZO-1, ZEB1, CD31, N-Cadherin, VE-Cadherin, Vimentin, α-SMA, Slug, Snail and TAGLN levels in HUVEC, MAEC and MPLEC treated with ox-LDL (100 μg/mL, 48 h). (Figure 2F, n = 3, Normalized to β-actin).

(G-I) Statistical analysis of PIM1, ZO-1, ZEB1, CD31, N-Cadherin, VE-Cadherin, Vimentin, α-SMA, Slug, Snail and TAGLN levels in HUVEC, MAEC and MPLEC treated with H_2O_2 (200 μM) and TGF-β (50 ng/mL, 48 h). (Figure 2F, n = 3, Normalized to β-actin).

Data are shown as mean ± SD. *P < 0.05, **P < 0.01, ***P < 0.001.

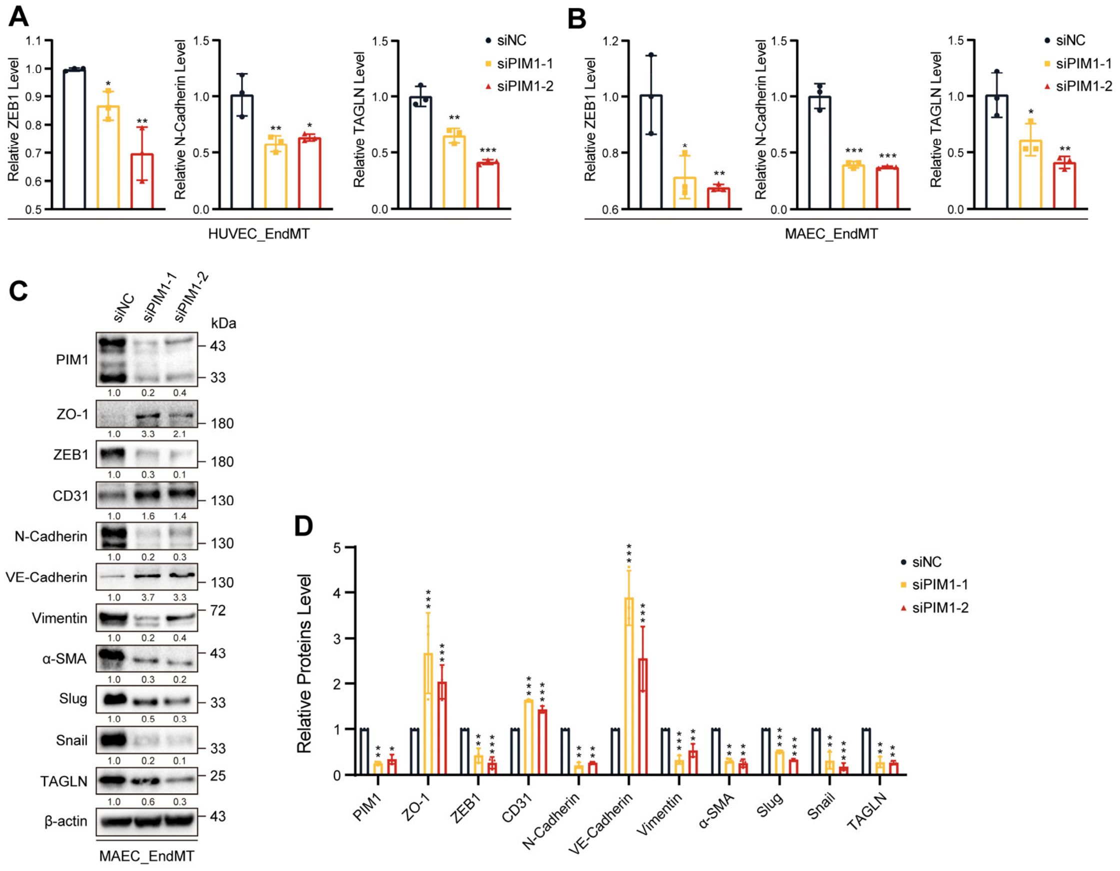

Figure S4. PIM1 Silence Attenuates the Process of EndMT.

(A) qRT-PCR analysis of ZEB1, N-Cadherin and TAGLN mRNA levels in HUVEC pretreated with siNC or siPIM1-1, siPIM1-2 and stimulated with H_2O_2 (200 μM) and TGF-β (50 ng/mL, 48 h).

(B) qRT-PCR analysis of ZEB1, N-Cadherin and TAGLN mRNA levels in MAEC pretreated with siNC or siPIM1-1, siPIM1-2 and stimulated with H2O2 (200 μM) and TGF-β (50 ng/mL, 48 h).

(C) Representative Western blot images and quantification of PIM1, ZO-1, ZEB1, CD31, N-Cadherin, VE-Cadherin, Vimentin, α-SMA, Slug, Snail and TAGLN levels in MAEC pretreated with siNC or siPIM1-1, siPIM1-2 and stimulated with H_2O_2 (200 μM) and TGF-β (50 ng/mL, 48 h).

(D) Statistical analysis of PIM1, ZO-1, ZEB1, CD31, N-Cadherin, VE-Cadherin, Vimentin, α-SMA, Slug, Snail and TAGLN levels in MAEC pretreated with siNC or

siPIM1-1, siPIM1-2 and stimulated with H_2O_2 (200 μM) and TGF-β (50 ng/mL, 48 h). (Figure S4C, n = 3, Normalized to β-actin).

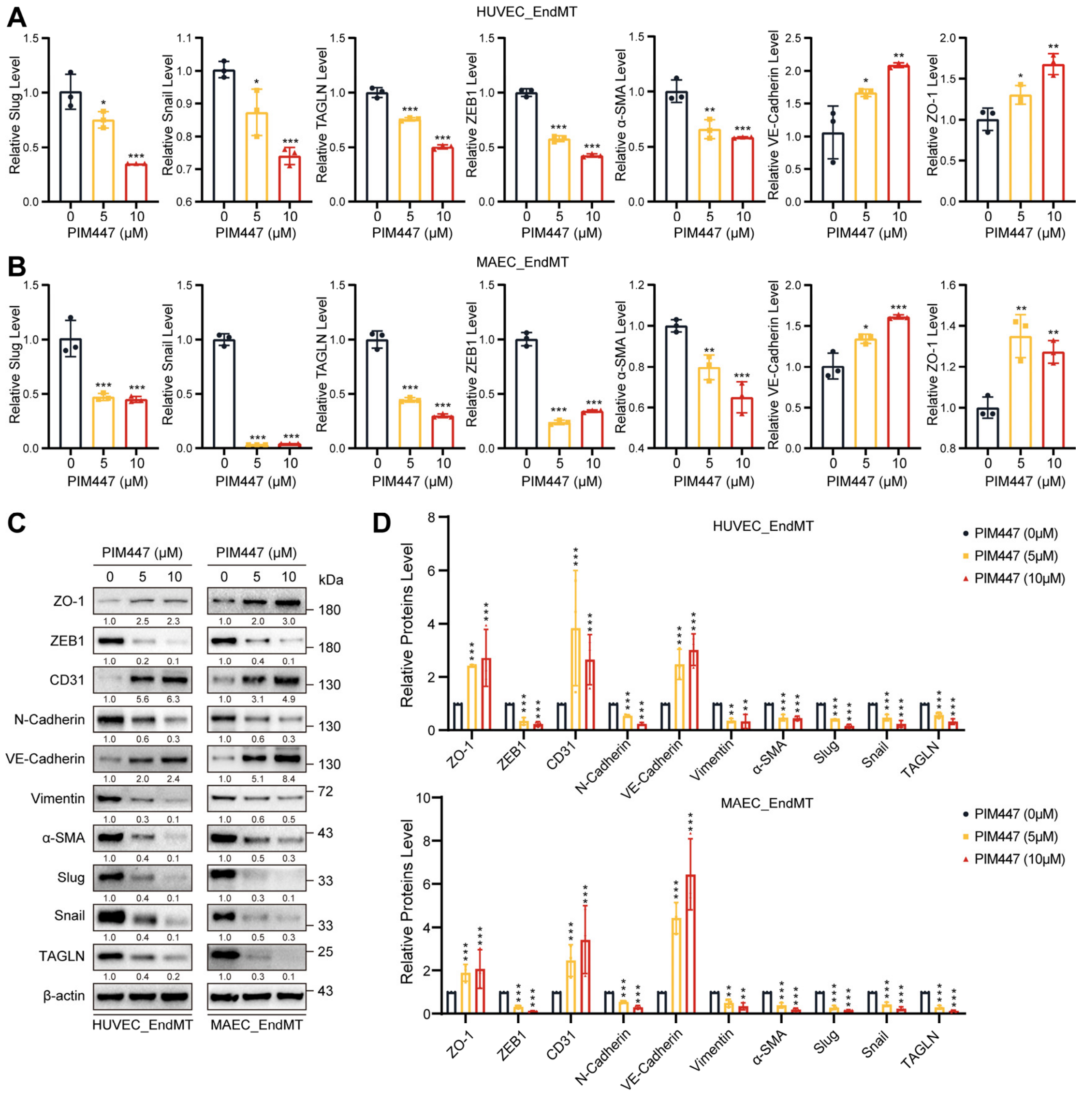

Figure S5. PIM447 Attenuates the Process of EndMT

(A) qRT-PCR analysis of SLUG, Snail, TAGLN, ZEB1, α-SMA, VE-Cadherin and ZO-1 mRNA levels in HUVEC stimulated with H_2O_2 (200 μM) and TGF-β (50 ng/mL, 48h) and treated with PIM447 (0, 5, 10 μM, 48 h).

(B) qRT-PCR analysis of SLUG, Snail, TAGLN, ZEB1, α-SMA, VE-Cadherin and ZO-1 mRNA levels in MAEC stimulated with H_2O_2 (200 μM) and TGF-β (50 ng/mL, 48 h) and treated with PIM447 (0, 5, 10 μM, 48 h).

(C) Representative Western blot images and quantification of ZO-1, ZEB1, CD31, N-Cadherin, VE-Cadherin, Vimentin, α-SMA, Slug, Snail and TAGLN levels in HUVEC

and MAEC stimulated with H_2O_2 (200 μM) and TGF-β (50 ng/mL, 48 h) and treated with PIM447 (0, 5, 10 μM, 48 h).

(D) Statistical analysis of ZO-1, ZEB1, CD31, N-Cadherin, VE-Cadherin, Vimentin, α-SMA, Slug, Snail and TAGLN levels in HUVEC and MAEC stimulated with H_2O_2 (200 μM) and TGF-β (50 ng/mL, 48 h) and treated with PIM447 (0, 5, 10 μM, 48 h). (Figure S5C, n = 3, Normalized to β-actin).

Data are shown as mean ± SD. *$P < 0.05$, **$P < 0.01$, ***$P < 0.001$.

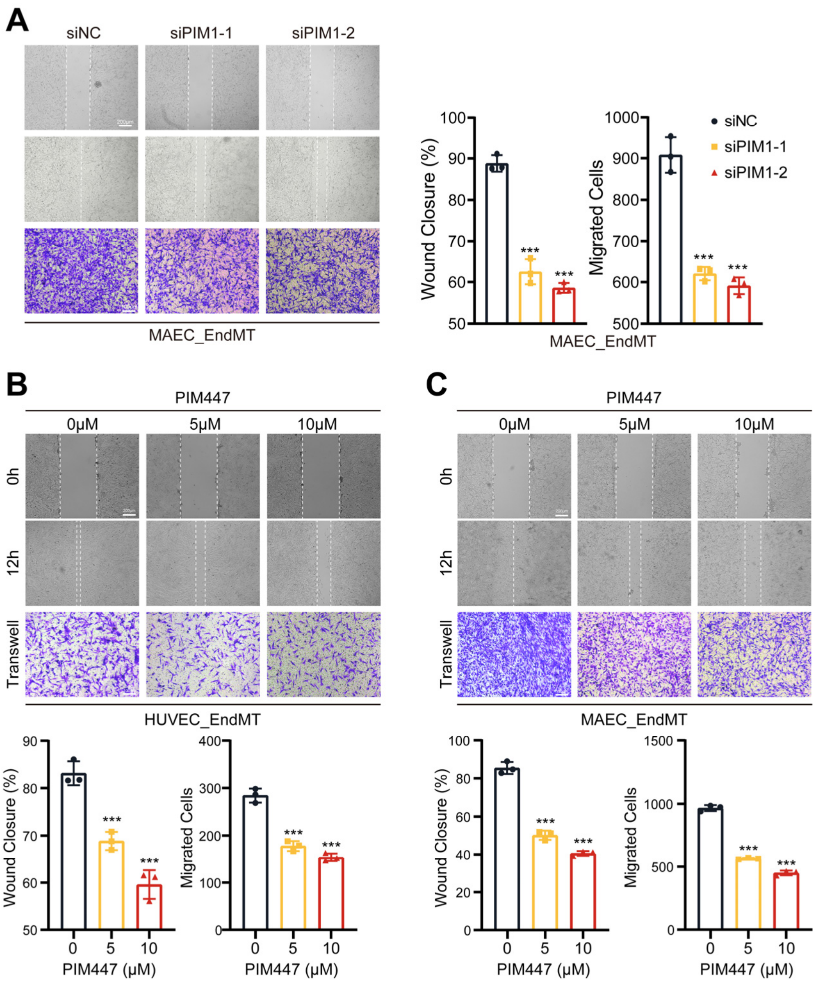

Figure S6. PIM447 Attenuates the Process of EndMT.

(A) Endothelial scratch wound healing assays and Transwell assay were performed, showing that PIM1 silenced attenuated migration of MAEC induced by H_2O_2 (200 μM) and TGF-β (50 ng/mL). Scale bar of wound healing assays = 400 μm. Scale bar of Transwell assay = 100 μm.

(B) Endothelial scratch wound healing assays and Transwell assay were performed, showing that PIM447 attenuated migration of HUVEC induced by H_2O_2 (200 μM) and TGF-β (50 ng/mL). Scale bar of wound healing assays = 200 μM. Scale bar of Transwell assay = 100 μm.

(C) Endothelial scratch wound healing assays and Transwell assay were performed, showing that PIM447 attenuated migration of MAEC induced by H_2O_2 (200 μM) and

TGF-β (50 ng/mL). Scale bar of wound healing assays = 200 μm. Scale bar of Transwell assay = 100 μm.

Data are shown as mean ± SD. $*P < 0.05$, $**P < 0.01$, $***P < 0.001$.

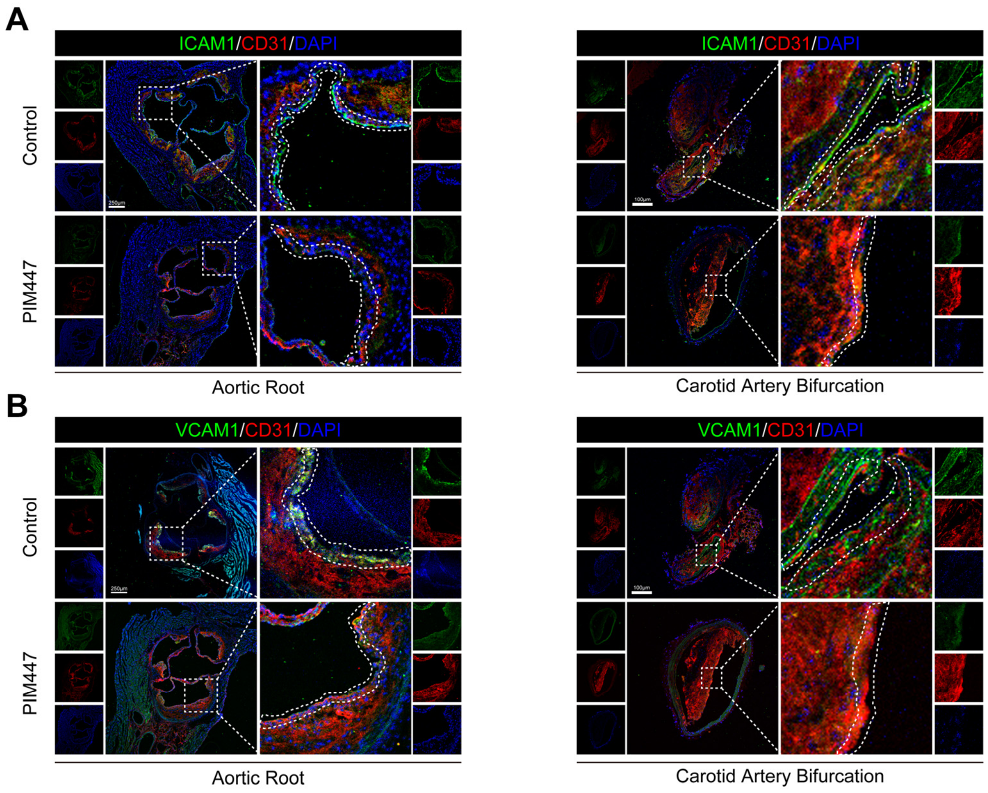

Figure S7. PIM447 inhibits the endothelial cell inflammation in vivo.

(A) Representative immunofluorescence staining images of ICAM1 expression in aortic root and carotid artery bifurcation sections in the 2 groups (n = 5). Scale bar of aortic root =250 μm. Scale bar of carotid artery bifurcation =100 μm.

(B) Representative immunofluorescence staining images of VCAM1 expression in aortic root and carotid artery bifurcation sections in the 2 groups (n = 5). Scale bar of aortic root =250 μm. Scale bar of carotid artery bifurcation =100 μm.

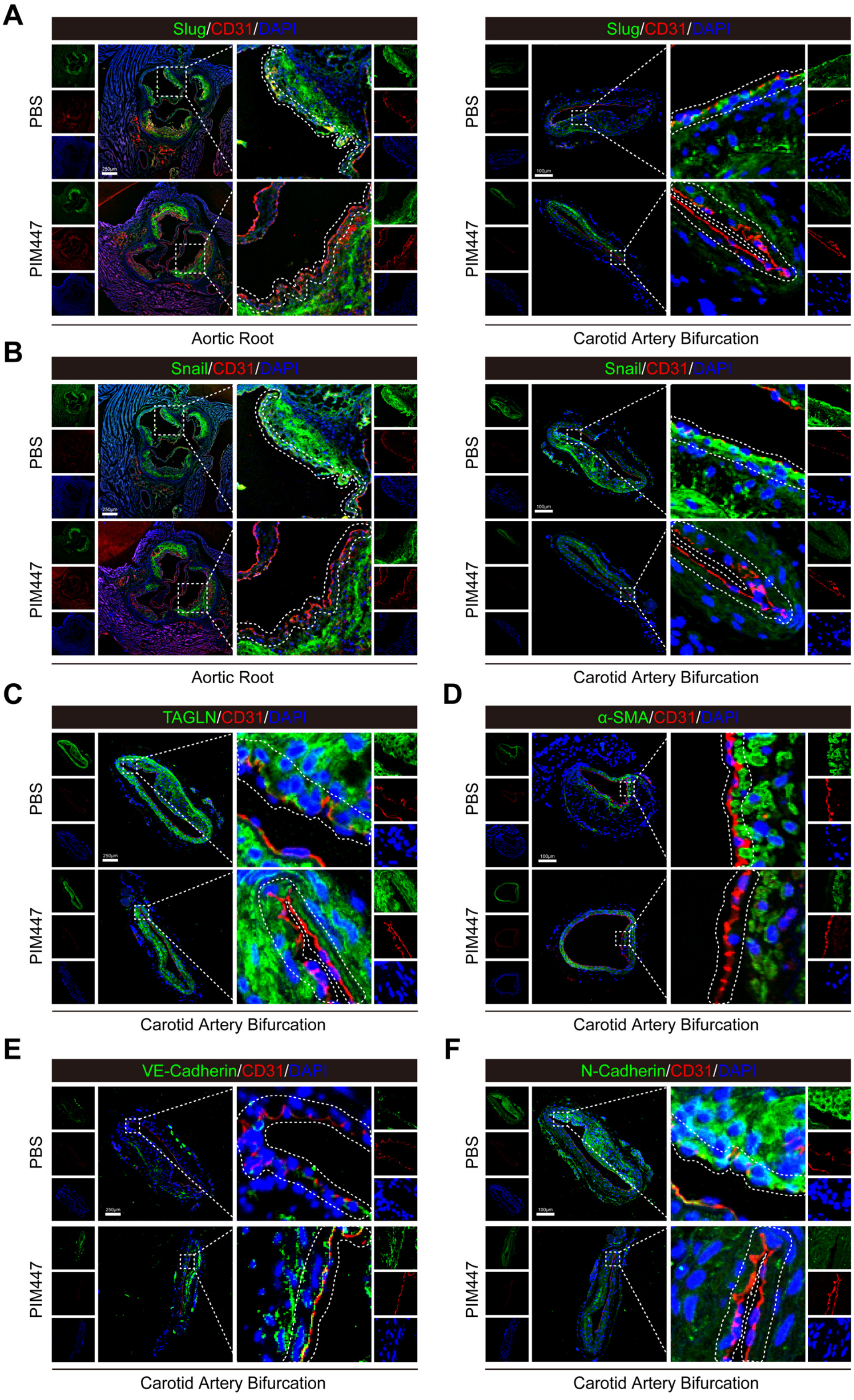
A
Slug/CD31/DAPI
PBS
PIM447
Aortic Root
Slug/CD31/DAPI
Carotid Artery Bifurcation
B
Snail/CD31/DAPI
Aortic Root
Snail/CD31/DAPI
Carotid Artery Bifurcation
C
TAGLN/CD31/DAPI
Carotid Artery Bifurcation
D
α-SMA/CD31/DAPI
Carotid Artery Bifurcation
E
VE-Cadherin/CD31/DAPI
Carotid Artery Bifurcation
F
N-Cadherin/CD31/DAPI
Carotid Artery Bifurcation

Figure S8. PIM447 Attenuates the process of EndMT in vivo.

(A) Representative immunofluorescence staining images of SLUG protein levels in aortic root and carotid artery bifurcation sections in the 2 groups (n = 5). Scale bar of aortic root =250 μm. Scale bar of carotid artery bifurcation =100 μm.

(B) Representative immunofluorescence staining images of Snail protein levels in aortic root and carotid artery bifurcation sections in the 2 groups (n = 5). Scale bar of aortic root =250 μm. Scale bar of carotid artery bifurcation =100 μm.

(C) Representative immunofluorescence staining images of TAGLN protein levels in carotid artery bifurcation sections in the 2 groups (n = 5). Scale bar =100 μm.

(D) Representative immunofluorescence staining images of α-SMA protein levels in carotid artery bifurcation sections in the 2 groups (n = 5). Scale bar =100 μm.

(E) Representative immunofluorescence staining images of VE-Cadherin protein levels in carotid artery bifurcation sections in the 2 groups (n = 5). Scale bar =100 μm.

(F) Representative immunofluorescence staining images of N-Cadherin protein levels in carotid artery bifurcation sections in the 2 groups (n = 5). Scale bar =100 μm.

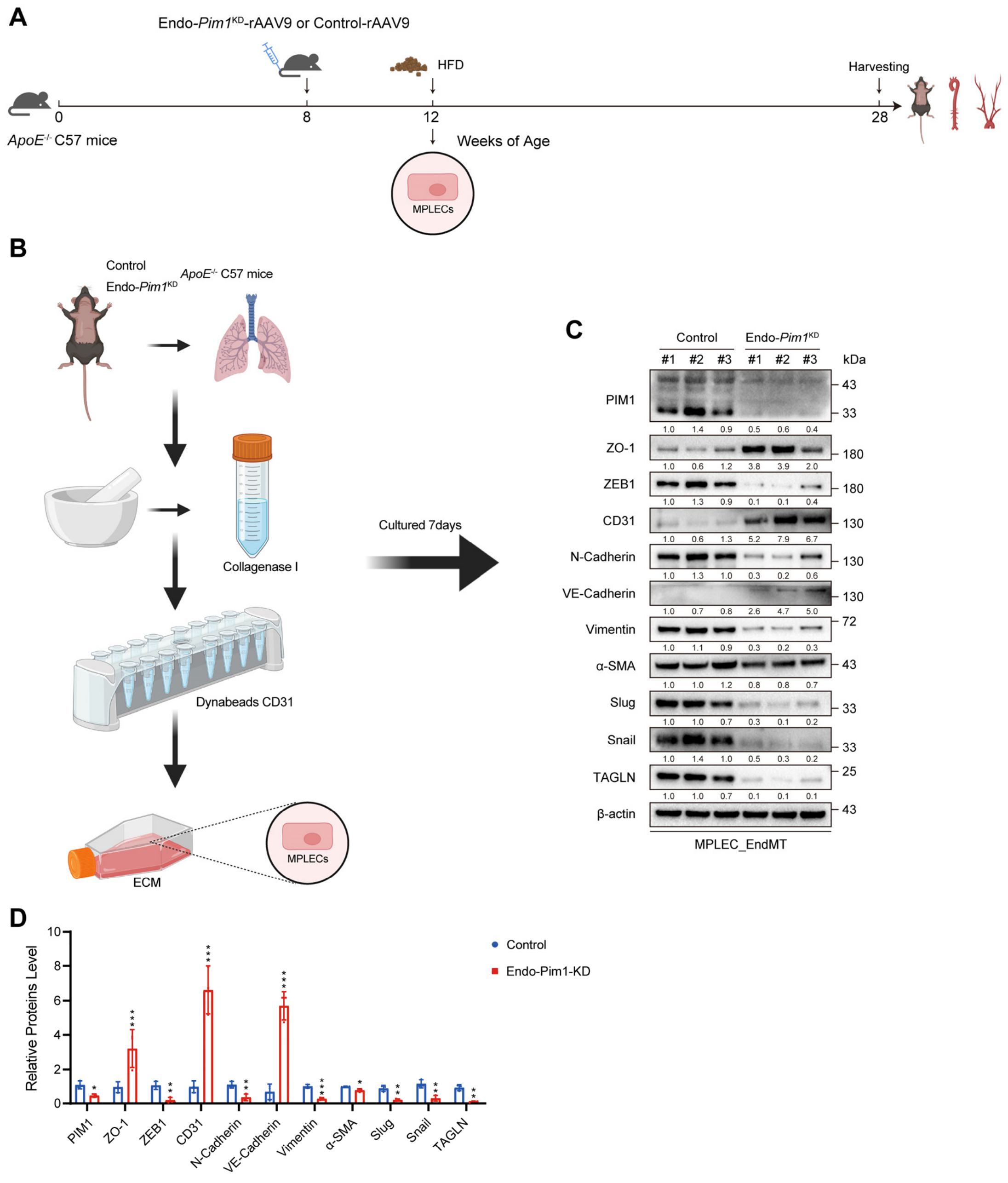

Figure S9. PIM1 Silence Attenuates the Process of EndMT

(A) Time axis of mouse model construction for atherosclerosis.

(B) Schematic diagram of the MPLEC extraction process.

(C) Representative Western blot images and quantification of PIM1, ZO-1, ZEB1, CD31, N-Cadherin, VE-Cadherin, Vimentin, α-SMA, Slug, Snail and TAGLN levels in MPLEC from 2 different group as indicated stimulated with H_2O_2 (200 μM) and TGF-β (50 ng/mL, 48 h).

(D) Statistical analysis of PIM1, ZO-1, ZEB1, CD31, N-Cadherin, VE-Cadherin, Vimentin, α-SMA, Slug, Snail and TAGLN levels in MPLEC from 2 different group as indicated stimulated with H_2O_2 (200 μM) and TGF-β (50 ng/mL, 48 h). (Figure S9C, n = 3, Normalized to β-actin).

Data are shown as mean ± SD. **P* < 0.05, ***P* < 0.01, ****P* < 0.001.

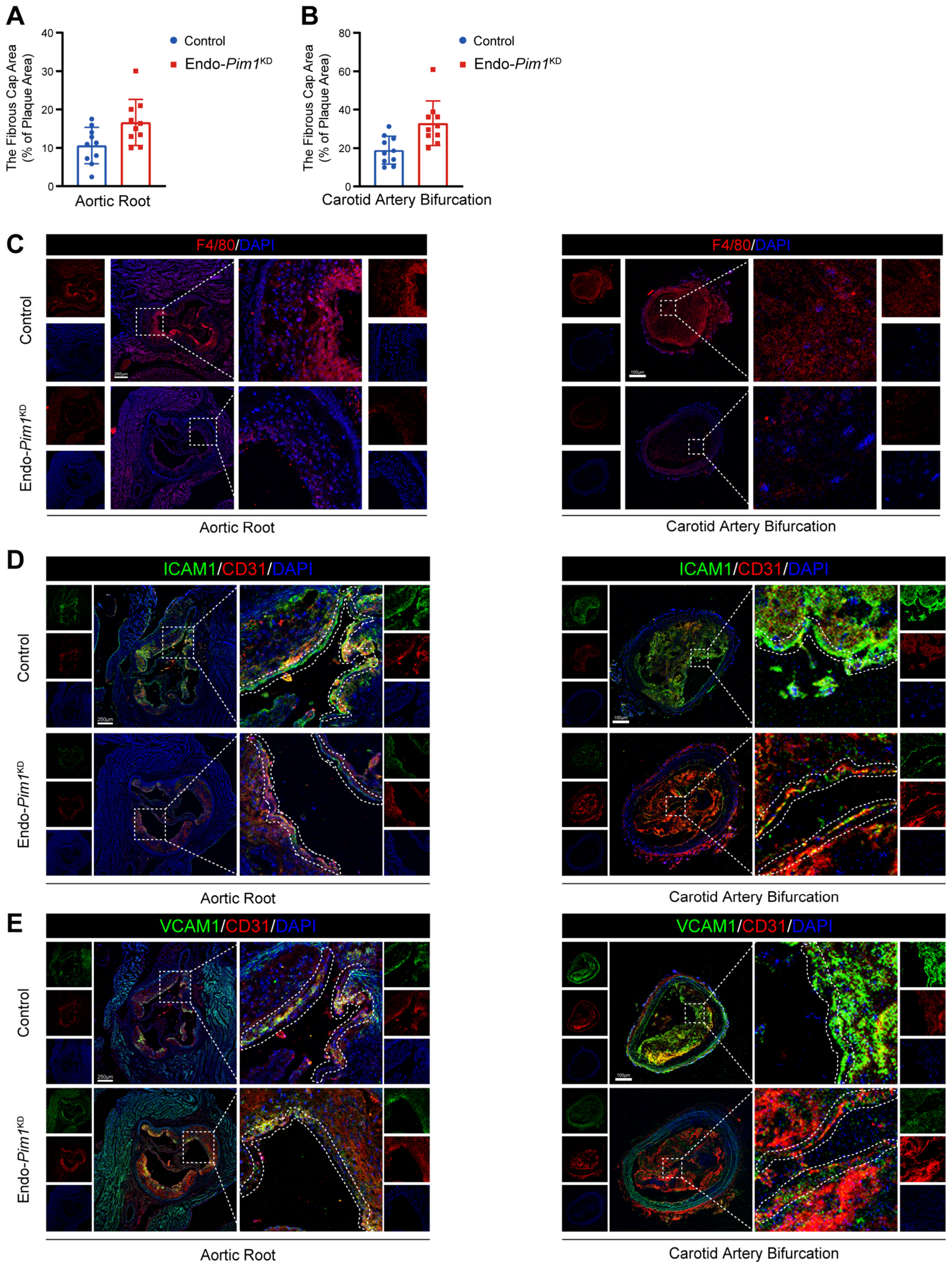

Figure S10. Endothelial Cell–Specific PIM1 Knockdown Reduces EndMT and Attenuates Atherosclerotic Plaque progress.

(A) Statistical analysis of the fibrous cap area in aortic root sections in the 2 groups (n = 10).

(B) Statistical analysis of the fibrous cap area in carotid artery bifurcation sections in the 2 groups (n = 10).

(C) Representative immunofluorescence staining images of F4/80 expression in aortic root and carotid artery bifurcation sections in the 2 groups (n = 10). Scale bar of aortic root =250 μm. Scale bar of carotid artery bifurcation =100 μm.

(D) Representative immunofluorescence staining images of ICAM1 expression in aortic root and carotid artery bifurcation sections in the 2 groups (n = 10). Scale bar of aortic root =250 μm. Scale bar of carotid artery bifurcation =100 μm.

(E) Representative immunofluorescence staining images of VCAM1 expression in aortic root and carotid artery bifurcation sections in the 2 groups (n = 10). Scale bar of aortic root =250 μm. Scale bar of carotid artery bifurcation =100 μm.

Data are shown as mean ± SD. **P* < 0.05, ***P* < 0.01, ****P* < 0.001.

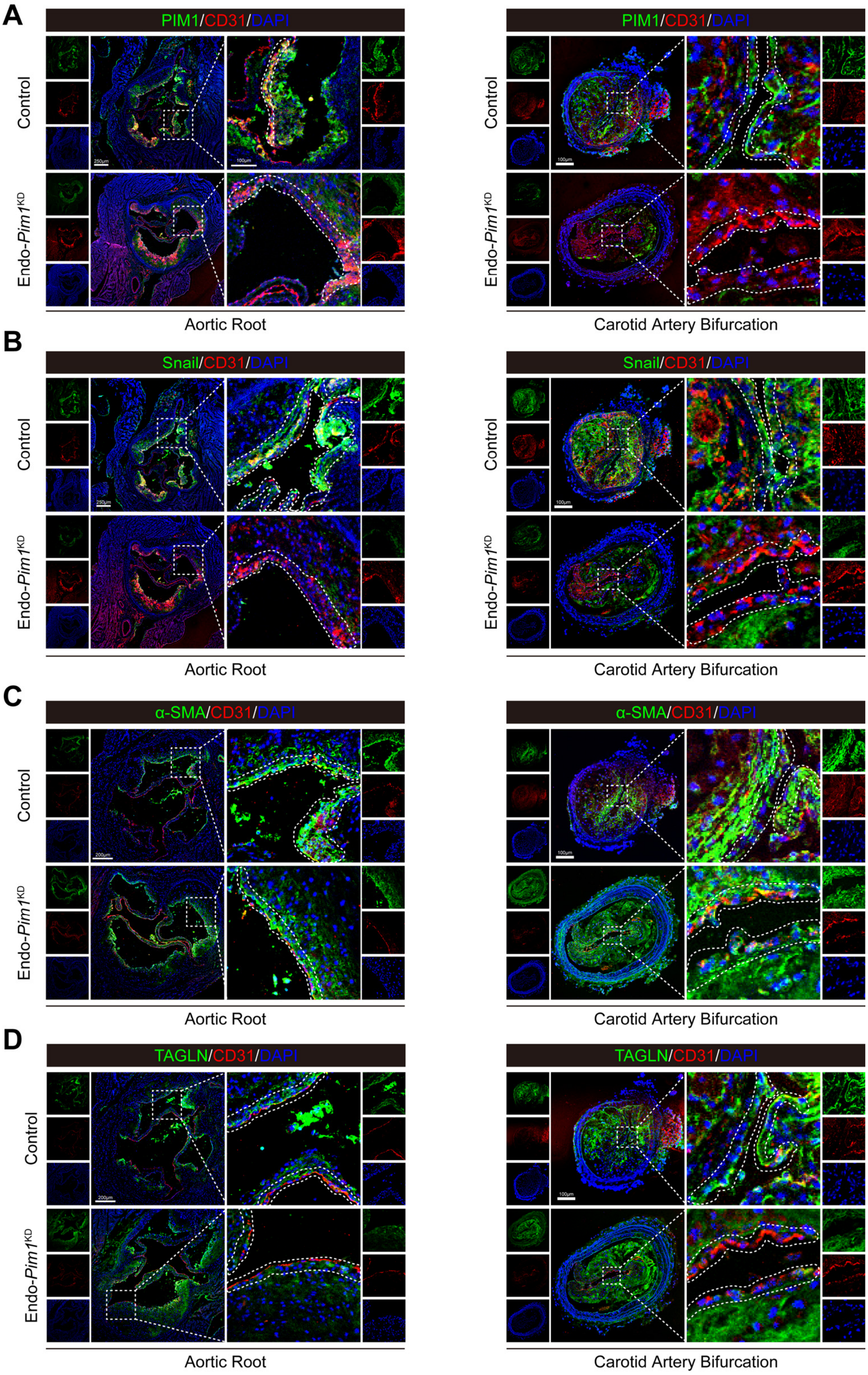
A
PIM1/CD31/DAPI
Control
Endo-Pim1KD
Aortic Root
Carotid Artery Bifurcation
B
Snail/CD31/DAPI
C
α-SMA/CD31/DAPI
D
TAGLN/CD31/DAPI

Figure S11. Endothelial Cell–Specific PIM1 Deletion Reduces EndMT.

(A) Representative immunofluorescence staining images of PIM1 protein levels in aortic root and carotid artery bifurcation sections in the 2 groups (n = 10). Scale bar of aortic root =250 μm. Scale bar of carotid artery bifurcation =100 μm.

.

(B) Representative immunofluorescence staining images of Snail protein levels in aortic root and carotid artery bifurcation sections in the 2 groups (n = 10). Scale bar of aortic root =250 μm. Scale bar of carotid artery bifurcation =100 μm.

(C) Representative immunofluorescence staining images of α-SMA protein levels in aortic root and carotid artery bifurcation sections in the 2 groups (n = 10). Scale bar of aortic root =250 μm. Scale bar of carotid artery bifurcation =100 μm.

(D) Representative immunofluorescence staining images of TAGLN protein levels in aortic root and carotid artery bifurcation sections in the 2 groups (n = 10). Scale bar of aortic root =250 μm. Scale bar of carotid artery bifurcation =100 μm.

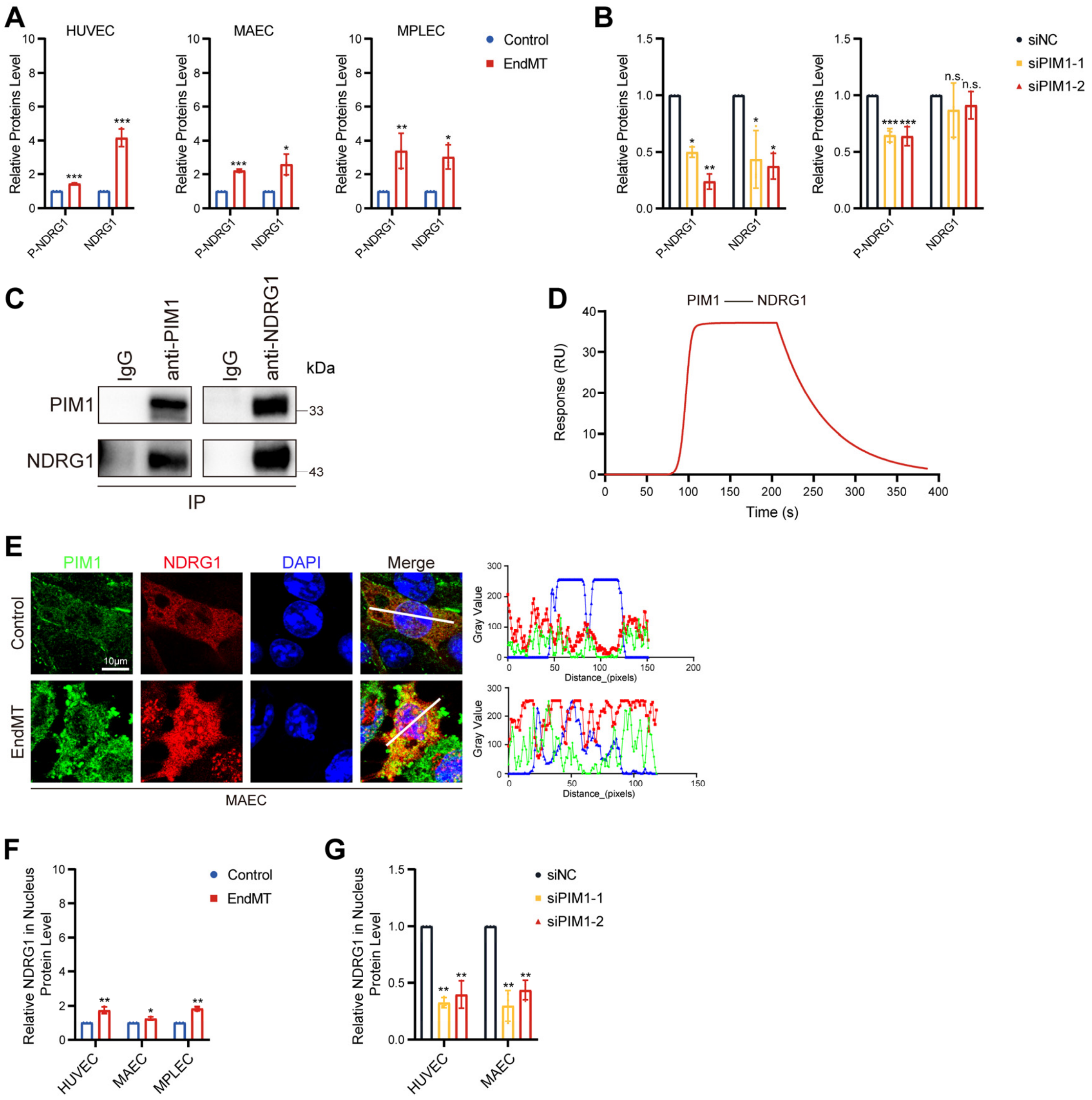

Figure S12. NDRG1 is Required for PIM1-Induced EndMT

(A) Statistical analysis of P-NDRG1 and NDRG1 levels in HUVEC, MAEC and MPLEC treated with H_2O_2 (200 μM) and TGF-β (50 ng/mL, 48 h). (Figure 6C, n = 3, Normalized to β-actin).

(B) Statistical analysis of P-NDRG1 and NDRG1 levels in HUVEC and MAEC pretreated with siNC or siPIM1-1, siPIM1-2 and stimulated with H_2O_2 (200 μM) and TGF-β (50 ng/mL, 48 h). (Figure 6D, n = 3, Normalized to β-actin).

(C) The Co-IP experiment detecting the interaction between PIM1 and NDRG1 in HUVEC treated with H_2O_2 (200 μM) and TGF-β (50 ng/mL, 48 h).

(D) Red lines represent the global interaction of PIM1 with NDRG1 in the SPR assay.

(E) Fluorescence colocalization and quantification between PIM1 (Green) and NDRG1 (Red) in MAEC. Scale bar = 10 μm.

(F) Statistical analysis of NDRG1 nuclear protein levels in HUVEC, MAEC and MPLEC treated with H_2O_2 (200 μM) and TGF-β (50 ng/mL, 48 h). (Figure 6K, n = 3, Normalized to H3).

(G) Statistical analysis of NDRG1 nuclear protein levels in HUVEC and MAEC pretreated with siNC or siPIM1-1, siPIM1-2 and stimulated with H_2O_2 (200 μM) and TGF-β (50 ng/mL, 48 h). (Figure 6L, n = 3, Normalized to H3).

Data are shown as mean ± SD. *$P < 0.05$, **$P < 0.01$, ***$P < 0.001$.

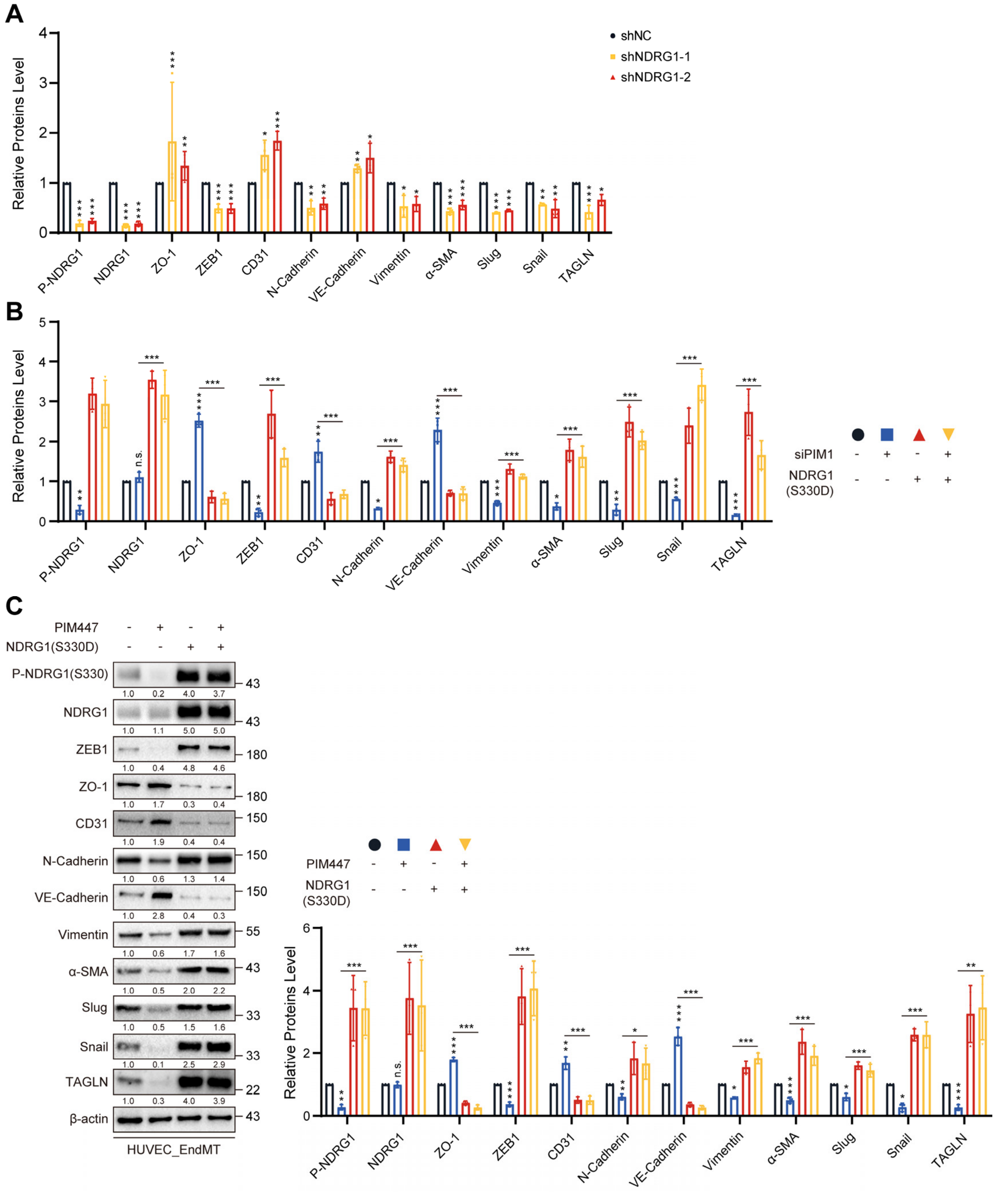

Figure S13. NDRG1 is Required for PIM1-Induced EndMT

(A) Statistical analysis of P-NDRG1, NDRG1, ZO-1, ZEB1, CD31, N-Cadherin, VE-Cadherin, Vimentin, α-SMA, Slug, Snail and TAGLN levels in HUVEC pretreated with shNC or shNDRG1-1, shNDRG1-2 and stimulated with H_2O_2 (200 μM) and TGF-β (50 ng/mL, 48 h). (Figure 7A, n = 3, Normalized to β-actin).

(B) Statistical analysis of P-NDRG1, NDRG1, ZO-1, ZEB1, CD31, N-Cadherin, VE-Cadherin, Vimentin, α-SMA, Slug, Snail and TAGLN levels in HUVEC pretreated as indicated and stimulated with H_2O_2 (200 μM) and TGF-β (50 ng/mL, 48 h). (Figure 7D, n = 3, Normalized to β-actin).

(C) Representative Western blot images and statistical quantification of P-NDRG1(S330), NDRG1, ZO-1, ZEB1, CD31, N-Cadherin, VE-Cadherin, Vimentin, α-SMA, Slug, Snail and TAGLN levels in HUVEC pretreated as indicated and stimulated with H_2O_2 (200 μM) and TGF-β (50 ng/mL, 48 h). (Figure S13C, n = 3, Normalized to β-actin).

Data are shown as mean ± SD. *$P < 0.05$, **$P < 0.01$, ***$P < 0.001$.

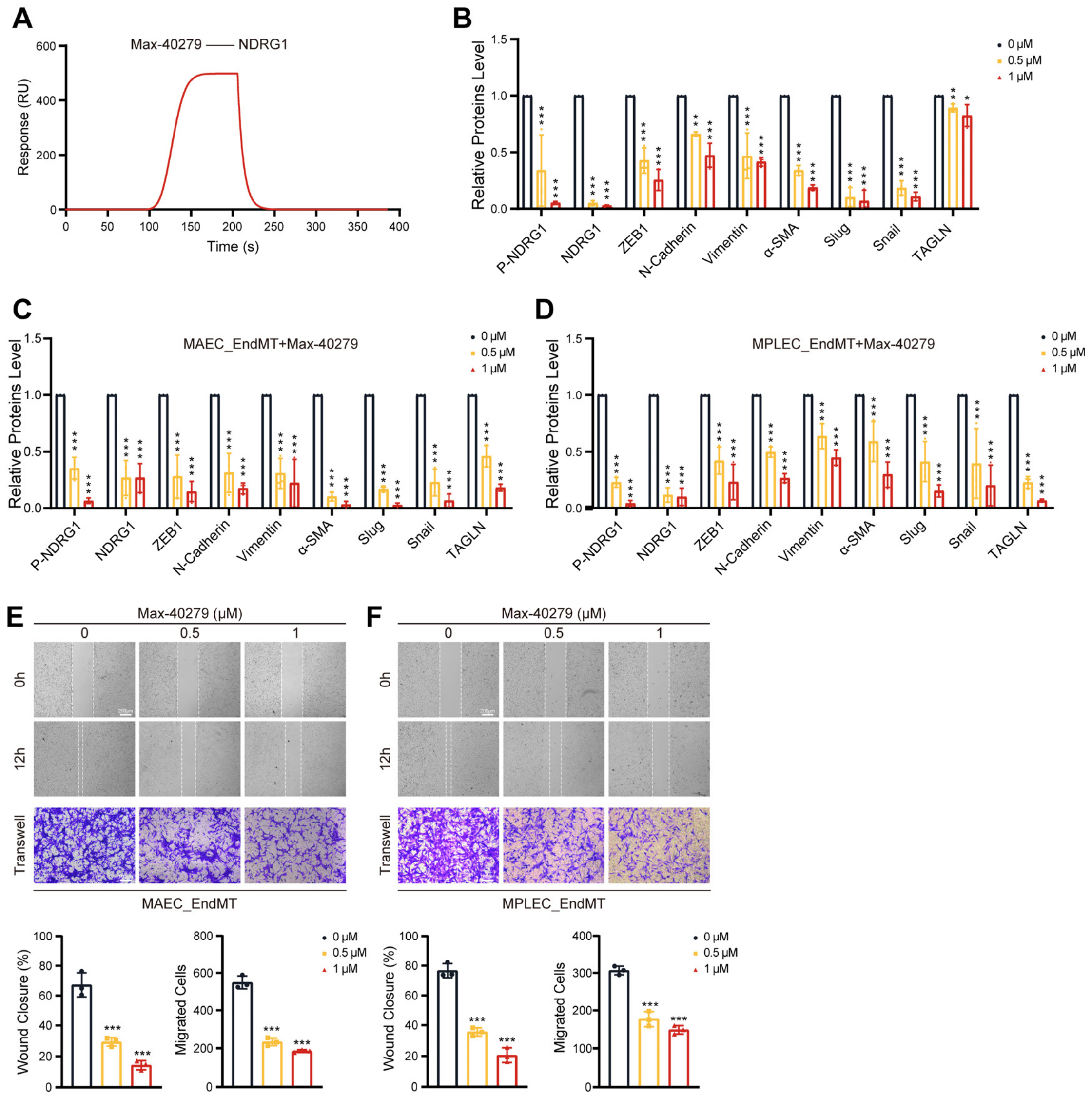

Figure S14. NDRG1 is Required for PIM1-Induced EndMT

(A) Red lines represent the global interaction of PIM1 with NDRG1 in the SPR assay.

(B-D) Statistical analysis of P-NDRG1, NDRG1, ZEB1, N-Cadherin, Vimentin, α-SMA, Slug, Snail and TAGLN levels in HUVEC, MAEC and MPLEC stimulated with H_2O_2 (200 μM) and TGF-β (50n g/mL, 48 h), and treated with Max-40279 (0.5 μM or 1 μM, 48 h). (Figure 7G, n = 3, Normalized to β-actin).

(E) Endothelial scratch wound healing assays and Transwell assay were performed, showing that Max-40279 attenuated migration of MAEC induced by H_2O_2 (200 μM) and TGF-β (50 ng/mL). Scale bar of wound healing assays = 200 μm. Scale bar of Transwell assay = 100 μm.

(F) Endothelial scratch wound healing assays and Transwell assay were performed, showing that Max40279 attenuated migration of MPLEC induced by H_2O_2 (200 μM) and TGF-β (50 ng/mL). Scale bar of wound healing assays = 200 μm. Scale bar of Transwell assay = 100 μm.

Data are shown as mean ± SD. **P* < 0.05, ***P* < 0.01, ****P* < 0.001.

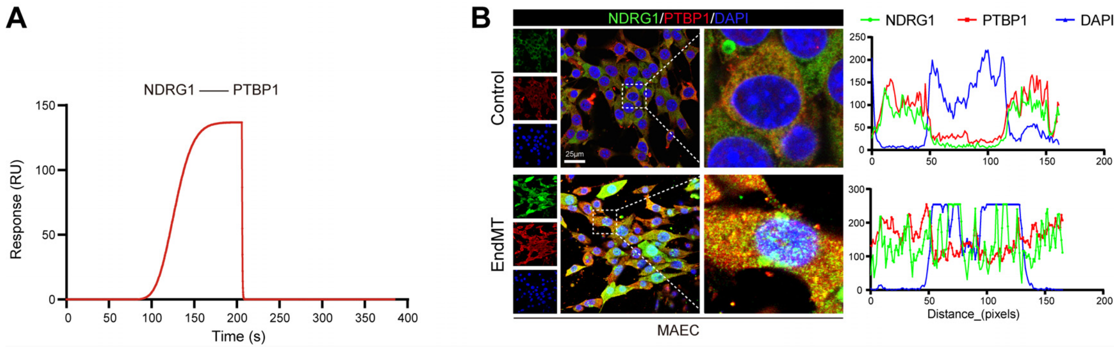

Figure S15. NDRG1 and PTBP1 collaborate to promote EndMT

(A) Red lines represent the global interaction of NDRG1 with PTBP1 in the SPR assay. (B) Fluorescence colocalization and quantification between NDRG1 (Green) and PTBP1 (Red) in MAEC. Scale bar = 25 μm.

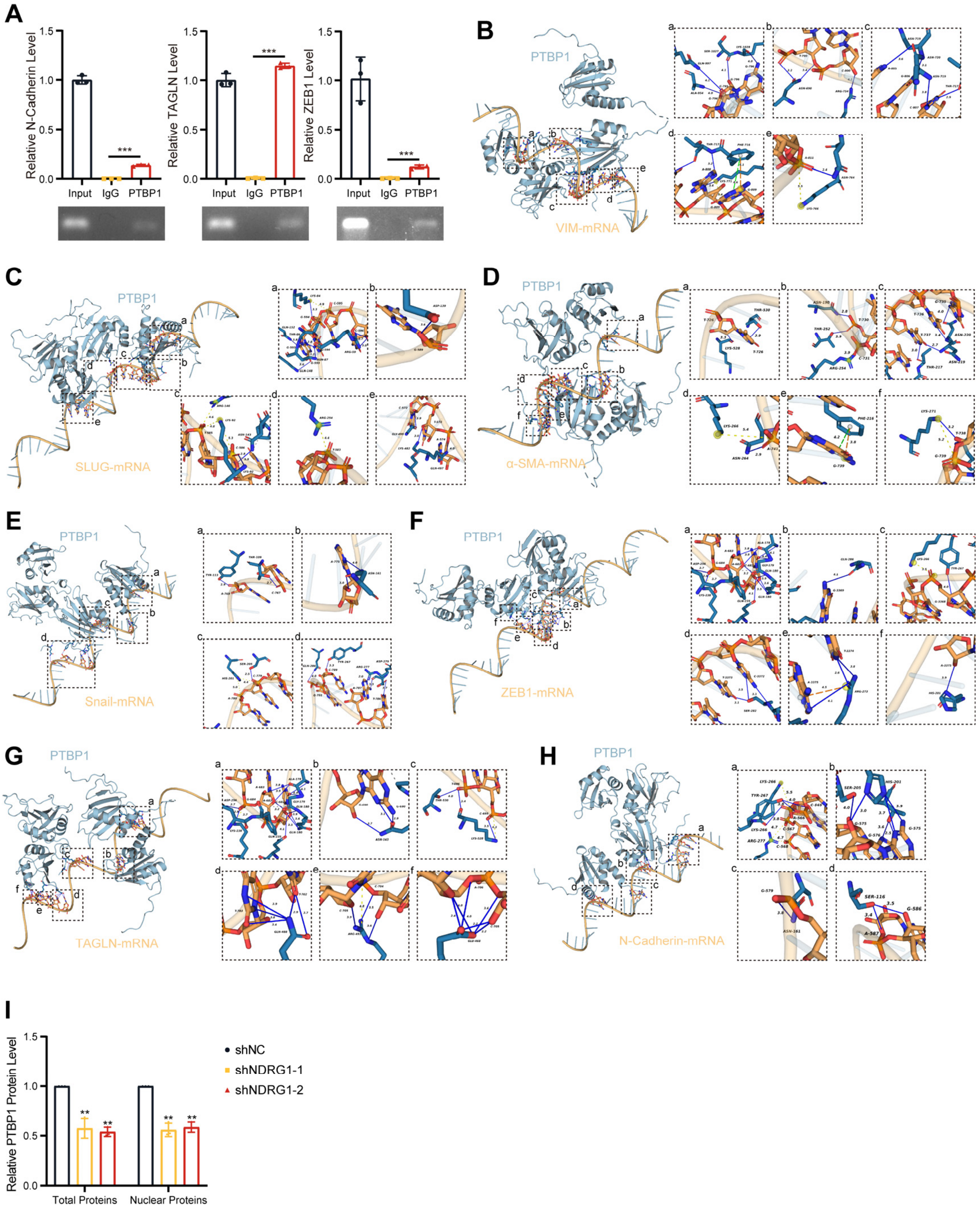

Figure S16. NDRG1 and PTBP1 collaborate to promote EndMT

(A) Immunopurification of PTBP1/RNA complexes or control experiments (IgG) from HUVEC cell extracts. qRT-PCR and agarose gel electrophoresis after reverse transcription and PCR detection the different mRNA level.

(B) The docking prediction of PTBP1 with Vimentin.

(C) The docking prediction of PTBP1 with SLUG.

(D) The docking prediction of PTBP1 with α-SMA.

(E) The docking prediction of PTBP1 with Snail.

(F) The docking prediction of PTBP1 with ZEB1.

(G) The docking prediction of PTBP1 with TAGLN.

(H) The docking prediction of PTBP1 with N-Cadherin.

(I) Statistical analysis of PTBP1 nuclear and total proteins levels in HUVEC pretreated with shNC or shNDRG1-1, shNDRG1-2 and stimulated with H_2O_2 (200 μM) and TGF-β (50 ng/mL, 48 h). (Figure 7J, K, n = 3, Normalized to β-actin and H3).

Data are shown as mean ± SD. *P < 0.05, **P < 0.01, ***P < 0.001.

Supplementary Tables

Table S1. Clinical data of Carotid atherosclerotic plaque patients involved in our study

Patient ID	Age at Surgery	Gender	Symptom	Plaque Status
21079150	65	Male	None	Stable
22029662	68	Female	None	Stable
22066510	57	Male	None	Stable
22074809	66	Male	None	Stable
22086769	66	Male	None	Stable
22099771	65	Male	None	Stable
22106659	60	Male	None	Stable
22155895	66	Male	None	Stable
22032335	74	Male	None	Stable
22087705	74	Female	None	Stable
22032484	74	Male	Yes	Unstable
22040294	64	Male	Yes	Unstable
22046392	67	Male	Yes	Unstable
22046504	58	Female	Yes	Unstable
22060724	74	Male	Yes	Unstable
22074849	71	Male	Yes	Unstable
22130905	69	Male	Yes	Unstable

22140380	72	Female	Yes	Unstable
22175444	74	Male	Yes	Unstable
23012420	68	Female	Yes	Unstable

Table S2. siRNA sequences used in this study

siRNA	Sequence (5'-3')
Homo-siPIM1-1	CAAGAUCUCUUCGACUUCATT
Homo-siPIM1-2	GGAUCCUGCUGUAUGAUAUTT
Mus-siPIM1-1	ACACAGUCUACACGGACUUTT
Mus-siPIM1-2	CCGAUAGUUUCGUGCUGAUTT

Table S3. shRNA sequences used in this study

shRNA	Sequence (5'-3')
Homo-Lenti-shNDRG1-1	GCCTACATCCTAACTCGATTT
Homo-Lenti-shNDRG1-2	CCTGGAGTCCTTCAACAGTTT
Mus-AAV9-shPim1	CCGAUAGUUUCGUGCUGAUTT

Table S4. Plasmids used in this study

Plasmids	Sequence (5'-3')
Homo-PIM1	Homo-Pim1-N-MYC in PCDNA3.1
Homo-NDRG1	Homo-NDRG1(NM_001135242.2)-C-HIS in PCDNA3.1
Homo-NDRG1(S330A)	Homo-NDRG1(S330A)-C-HIS in PCDNA3.1
Homo-NDRG1(S330D)	Homo-NDRG1(S330D)-C-HIS in PCDNA3.1
Homo-NDRG1(del:180-294aa)	Homo-NDRG1(del:180-294aa)-C-HIS in PCDNA3.1
Homo-NDRG1(del:326-350aa)	Homo-NDRG1(del:326-350aa)-C-HIS in PCDNA3.1

Table S5. qRT-PCR primers used in this study

Gene	Forward (5'-3')	Reverse (5'-3')
Homo-PIM1	AAAATCAACTCGCTTGCCCAC	CTGAGTAGACCGAGCCGAAG
Homo-ZEB1	AATTCACAGTGGAGAGAAGCCA	GGTCGCCCATTCACAGGTAT
Homo-ZO-1	TCACGCAGTTACGAGCAAGT	TGAAGGTATCAGCGGAGGGA
Homo-VE-Cadherin	GCATCGGTTGTTCAATGCGT	CGCTTCCACCACGATCTCAT
Homo-N-Cadherin	TCCTGCTTATCCTTGTGCTGA	AAAAGTTGTTTGGCCTGGCG
Homo-CD31	GCTGACCCTTCTGCTCTGTT	ATCTGGTGCTGAGGCTTGAC
Homo-α-SMA	ATGCCTCTGGACGCACAACT	CCCGGACAATCTCACGCTCA
Homo-Slug	GAGCATACAGCCCCATCACT	CTCACTCGCCCCAAAGATGA
Homo-Snail	GACCCCAATCGGAAGCCTAA	AGGGCTGCTGGAAGGTAAAC
Homo-TAGLN	TGAAGGCGGCTGAGGACTAT	ATCTCCACGGTAGTGCCCAT
Homo-β-actin	GAAGAGCTACGAGCTGCCTGA	CAGACAGCACTGTGTTGGCG
Homo-NDRG1	CGACCTGGAGATTGAGCGAC	CCGCCATCTTGAGGAGAGTG
Homo-	TACAAAGCGGGGATCTGACG	TCGGCTGTCACCTTTGAACT

PTBP1		
Mus-PIM1	TGGATTCGCTACCATCGCTAC	TCTTCATCGTGCTCAAACGGA
Mus-ZEB1	GGGACCTCAATGCACTTCCA	GTGGCTGACTGGGAGACAAA
Mus-ZO-1	GATTTACCCGTCAGCCCTTCT	TGGGCCTAAGTATCCCGTCT
Mus-VE-Cadherin	AAGACATCCGAGTGGGCAAG	CTGTACTCGCCCTGCATGAT
Mus-N-Cadherin	GGCAATCCCACTTATGGCCT	TCCGTGACAGTTAGGTTGGC
Mus-CD31	GCATCGGCAAAGTGGTCAAG	GGGTGCAGTTCCATTTTCGG
Mus-α-SMA	TTCGTGACTACTGCCGAGC	GTCAGGCAGTTCGTAGCTCT
Mus-Slug	AGAAGCCCAACTACAGCGAA	ATAGGGCTGTATGCTCCCGA
Mus-Snail	ACCCTCATCTGGGACTCTCTC	CAGCGAGGTCAGCTCTACG
Mus-TAGLN	TGGCTGTGACCAAAAACGATG	TGCTCCTGGGCTTTCTTCATA
Mus-β-actin	GGCTGTATTCCCCTCCATCG	CCAGTTGGTAACAATGCCATGT
Mus-NDRG1	ACAAGACCTGCTACAACCCC	AGCCCAAACTGGTGAAGGAC

Table S6. Antibodies used in this study

Antibodies	Manufacturer	Cat No.	Applications
β-actin	Proteintech	66009-1-Ig	WB
PIM1	Abcam	ab300453	WB, IP
PIM1	Santa	sc-374116	IF, IHC
CD31	Servicebio	GB11063-2-100	WB, IF
CD31	Servicebio	GB12063-100	IF
CD31	Servicebio	GB12064-100	IF
ZO-1	Thermo Fisher	33-9100	WB
ZEB1	CST	70512S	WB
N-Cadherin	Proteintech	22018-1-AP	WB
N-Cadherin	Proteintech	66219-1-Ig	IF
VE-Cadherin	CST	2500S	WB
VE-Cadherin	Proteintech	66804-1-Ig	IF, IHC
Vimentin	Proteintech	10366-1-AP	WB
α-SMA	Proteintech	67735-1-Ig	WB
α-SMA	Abcam	ab7817	IF
Slug	CST	9585S	WB
Slug	Santa	sc-166476	IF, IHC
Snail	CST	3879S	WB
Snail	Santa	sc-271977	IF, IHC

TAGLN	Proteintech	10493-1-AP	WB
TAGLN	Proteintech	60213-1-Ig	IF
NDRG1	Abcam	ab124689	WB, IF, IP
NDRG1(phospho S330)	Abcam	ab124713	WB
Histone H3	CST	9715S	WB
PTBP1	Proteintech	67462-1-Ig	WB
PTBP1	Proteintech	12582-1-AP	WB, IF, IP, RIP
Myc-Tag	CST	2276S	IP
6*His-Tag	Proteintech	10001-0-AP	WB
6*His-Tag	Proteintech	66005-1-Ig	IP

Table S7. The identified proteins interacted with PIM1 by LC–MS/MS analysis

MS4A10	ACTB	EEF1A2	IMPDH2	ABCA5
NDRG1	MYH9	MYH10	S100A8	IGKV4-1
KRT1	KRT2	KRT5	KRT16	TFAM
SNPH	STAG2	VIM	IGKC	TMPO
KRT10	NUDCD2	KRT14	KRT17	KRT6B
KRT9	PIM1	MYL6	H1-3	ACTA1
ALB	IGKV2-29	TUBA3D	HRNR	TUBB
CACNB2	DCD			

Table S8 The top five small molecule drugs with NDRG1 have the lowest binding energies

No.	Name	Cas ID	Binding energies
4159	N-[2-(Carbamimidamidooxy)ethyl]-2-{6-Cyano-3-[(2,2-Difluoro-2-Pyridin-2-Ylethyl)amino]-2-Fluorophenyl}acetamide	-	-5
4059	2-(6-Chloro-3-{[2,2-difluoro-2-(2-pyridinyl)ethyl]amino}-2-oxo-1(2H)-pyrazinyl)-N-[(2-fluoro-6-pyridinyl)methyl]acetamide	312904-60-2	-4.4
7482	MAX-40279	2070931-57-4	-4.4
6905	Naftazone	15687-37-3	-4.2
7802	Stenoparib	1140964-99-3	-4.2

Table S9. The identified proteins interacted with NDRG1 in nuclear proteins by LC–MS/MS analysis

Gene ID	Protein ID	Peptides	Score	Abundances
MYH8	P13535	2	4.08	78104912.0
ADAMTSL4	Q6UY14	1	2.33	40128336.0
TBKBP1	A7MCY6	1	3.24	39052456.0
TUBA3D	P0DPH8	5	18.92	4632357.625
MBP	P02686	2	5.55	3938560.84375
GRIA1	P42261	1	0.0	3080987.0
PLP1	P60201	3	6.89	2602124.1875
TUBB	P07437	7	13.04	2114089.625
NDRG1	Q92597	3	12.88	1801817.34375
RPL27A	P46776	1	2.3	1240122.625
PARP1	P09874	3	7.02	1188897.90625
ATP1A2	P50993	5	10.52	959354.015625
IGLL5	B9A064	1	2.29	944982.0
HNRNPK	P61978	2	4.65	867004.40625
H2AZ2	Q71UI9	1	7.22	831455.9375
LMNB1	P20700	3	7.59	775806.375
HNRNPH1	P31943	4	9.72	755502.46875
ATP5F1A	P25705	3	6.67	686215.921875
NPM1	P06748	1	2.31	634930.625
RAN	P62826	1	2.03	623162.9375
YWHAZ	P63104	2	3.93	596841.3125
RPS13	P62277	1	2.13	483881.71875
RBMX	P38159	1	1.95	444043.5625
ATP5F1B	P06576	2	7.09	388387.921875
RPS8	P62241	1	2.14	374163.3125
RPS23	P62266	1	1.99	373401.625
SRSF7	Q16629	1	0.0	359095.53125
DHX9	Q08211	1	5.38	349699.625
NCL	P19338	1	1.92	345739.78125
SAFB	Q15424	1	2.12	341473.28125
HSPA7	P48741	1	2.15	336358.9375
PTBP1	P26599	1	1.98	318917.15625

Gene ID	Protein ID	Peptides	Score	Abundances
RPL29	P47914	1	1.91	311830.625
HSP90AA1	P07900	1	1.97	305896.53125
CKB	P12277	1	2.1	293870.84375
LBR	Q14739	1	0.0	285852.0625
ILF2	Q12905	1	0.0	285054.875
ALDOA	P04075	1	2.24	257677.609375
RPL7	P18124	1	2.19	221118.421875
TMPO	P42167	1	1.99	210581.328125
ILF3	Q12906	1	2.06	201099.25
RPL8	P62917	1	2.31	199844.5625
DPYSL2	Q16555	1	2.95	198080.390625
SRSF8	Q9BRL6	1	2.24	196025.046875
LMNA	P02545	1	2.06	192719.828125
MDH2	P40926	1	2.04	192424.140625
H1-10	Q92522	1	1.92	148243.59375
RCC1	P18754	1	2.35	136217.640625
RNASE4	P34096	1	2.54	0
PKM	P14618	1	2.48	0
H2AC1	Q96QV6	1	3.63	0
RPL13	P26373	1	2.53	0
QRICH1	Q2TAL8	1	0.0	0
